# Thermogenic methane beneath the North Greenland Ice Sheet revealed by isotopic and geological evidence

**DOI:** 10.1038/s41467-026-75951-4

**Published:** 2026-07-24

**Authors:** M. Ketzer, M. Jakobsson, K. Faehnrich, C. H. Akhoudas, J. Prytherch, C. Chang, C. Yu, M. Sundberg, H. Drake, J. Lattaud, W-L Hong, M. O’Regan, N. Kirchner, C. Stranne

**Affiliations:** 1 Centre for the Environment (CENWIN), Department of Biology and Environmental Science, Linnaeus University, Kalmar, Sweden; 2 Department of Geological Sciences, Stockholm University, Stockholm, Sweden; 3 Bolin Centre for Climate Research, Stockholm University, Stockholm, Sweden; 4 College of Science, Adelaide University, Adelaide, SA Australia; 5 Department of Earth Sciences, Uppsala University, Uppsala, Sweden; 6 Department of Environmental Science, Stockholm University, Stockholm, Sweden; 7 Baltic Sea Centre, Stockholm University, Stockholm, Sweden; 8 Department of Physical Geography, Tarfala Research Station, Stockholm University, Stockholm, Sweden

**Keywords:** Solid Earth sciences, Biogeochemistry, Climate sciences

## Abstract

Glacial meltwater has been increasingly recognised as a potential source of atmospheric methane, yet its origin and variability in the High Arctic remain poorly constrained. In this study, we present measurements of methane concentration and carbon‑isotope composition in meltwater draining the northern Greenland Ice Sheet. Here we show that methane concentrations (12-20 nM) are significantly lower than those reported from other Greenland catchments, despite clear evidence of subglacial input. Isotopic signatures and regional geological context indicate that this methane is predominantly thermogenic, reflecting a geological source that is largely independent of subglacial microbial activity. These findings advance current understanding of methane sources beneath the Greenland Ice Sheet, revealing a thermogenic contribution alongside microbial methane in Arctic methane cycling. In this work we highlight the need to account for geological methane reservoirs when assessing present and future cryosphere–carbon feedbacks.

## Abstract (web summary)

This study shows that methane beneath the North Greenland Ice Sheet derives from deep geological sources and, unlike microbial methane found elsewhere in Greenland, it is largely independent of surface biogeochemical processes.

## Introduction

Methane released from beneath ice sheets has recently emerged as a potentially important component of the global carbon cycle, particularly as ice sheets retreat^1^. Most current understanding of subglacial methane production beneath the Greenland Ice Sheet (GrIS) is based on observations from its southern and western margins, where methane is predominantly microbial in origin^2–7^. However, these datasets represent only a small fraction of the ice sheet, and the absence of measurements from northern Greenland limits our ability to evaluate spatial variability in methane sources, fluxes, and climatic relevance at the ice‑sheet scale.

Direct measurements of methane beneath the GrIS are still relatively recent, with the first observations from subglacial meltwaters published just over a decade ago^6,8,9^. Subsequent studies in the southwestern GrIS revealed high dissolved methane concentrations and potentially significant atmospheric emissions, in some cases rivalling fluxes from major global rivers^3^. Conversely, meltwaters from South Greenland display methane concentrations close to atmospheric equilibrium^5,7^. A recent regional study encompassing most of western Greenland reported methane concentrations in subglacial and proglacial waters spanning four orders of magnitude^7^. These contrasts highlight substantial regional heterogeneity but offer little insight into the North GrIS, one of the most understudied sectors of the ice sheet.

The North GrIS overlies sedimentary basins that host petroleum‑bearing formations and documented natural oil and gas seeps^10,11^, conditions distinct from the crystalline bedrock that dominates the southern and southwestern GrIS where microbial methanogenesis dominates^3,5,6^. Such geological settings raise the possibility that deep thermogenic methane contributes to subglacial methane budgets, yet this source is largely absent from current conceptual and numerical models^2^. Furthermore, rapid retreat of northern glaciers may reactivate underlying tectonic structures, potentially enhancing geological degassing during deglaciation^12–15^.

This region, therefore, provides a critical setting to determine whether subglacial methane beneath the GrIS is universally governed by microbial processes or whether geological sources exert a previously unrecognised influence. Resolving this distinction is essential for constraining present and future methane budgets beneath large ice masses, with implications extending beyond Greenland to other glaciated sedimentary basins, including parts of the Canadian High Arctic and Antarctica.

**Fig. 1:**
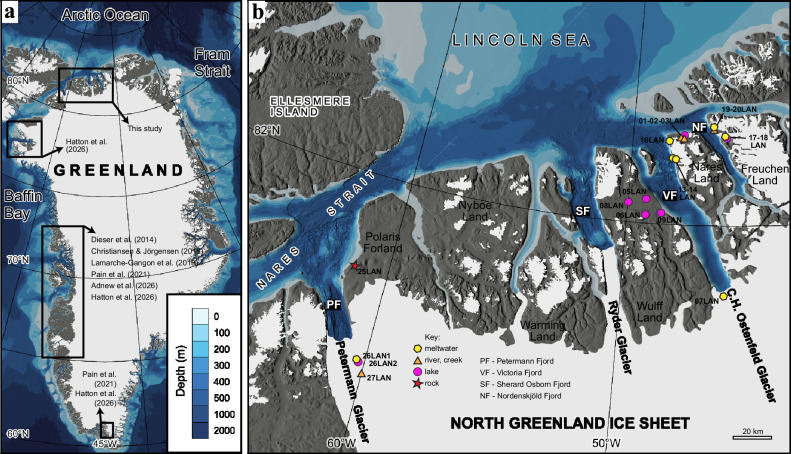
Location map of the study area and sampling points. **a** Areas in south, southwestern, and northwestern Greenland with previous methane data used in this paper and the present study area in the north. **b** Detail of the study area showing location of sampling stations for meltwater (yellow hexagons), rivers and creeks (orange triangles), lakes (purple circles), and rock (red star). Note that sampling locations 26LAN2 and 27LAN, adjacent to Petermann Glacier, correspond to a lake and a creek, respectively. These features cannot be visually distinguished from the ice sheet at the map scale. Coordinates for all sampling sites are in Supplementary Table 1. Bathymetric data are from the International Bathymetric Chart of the Arctic Ocean (IBCAO) Version 5.0^46^, which incorporates BedMachine Version 5 data for the inner-fjord bathymetry and subglacial topography^47^. The bathymetry of Victoria Fjord and Nordenskjöld Fjord has been updated using data collected during the GEOEO 2024 expedition^17^.

In August and September 2024, during the GEOEO-North of Greenland 2024 Expedition, icebreaker Oden visited the far North of Greenland and the Lincoln Sea, and was the first marine research vessel to enter Victoria Fjord^16,17^. Through helicopter reconnaissance, strategic terrestrial sites were selected for sampling meltwaters (i.e., waters running directly underneath glaciers) in the northern part of the GrIS, in the vicinity of the Petermann, Sherard Osborn, Victoria, and Nordenskjöld fjords (Fig. 1). We also sampled waters of periglacial lakes (with and without clear connection with glacial runoff), creeks and rivers (all connected to glacial runoff), as well as an exposed limestone with oil stains for comparison (Fig. 1; Supplementary Notes; Supplementary Figs. 1 and 2).

Oil seeps were previously described in several locations in the area (Supplementary Fig. 2)^11^. Gas seeps are more difficult to detect, but mid-water sonar images, acquired during a previous expedition in 2019^18^, revealed the existence of submarine gas flares in the Sherard Osborn and Petermann fjords, and Nares Strait, similar to those recently identified in northeastern Greenland^19^. The existence of the flares, many aligned to regional structures^18^, supports the hypothesis of ongoing degassing of the petroleum-rich Palaeozoic bedrock, potentially amplified by tectonism caused by glacier melt and isostatic rebound^12,13^.

In this work, we show that methane exported from the studied northern GrIS catchment is compatible with a thermogenic origin, in contrast to the microbial signatures reported for other GrIS catchments^3–7^. Unlike microbial methane, thermogenic methane originates from a deep geological carbon reservoir that can be mobilised independently of contemporary biological activity beneath the ice sheet^20^. As glacier retreat accelerates, such processes may enhance the transfer of long‑stored methane from the subsurface to surface environments and potentially to the atmosphere^14,21^.

## Results and discussion

### Methane concentration

**Fig. 2:**
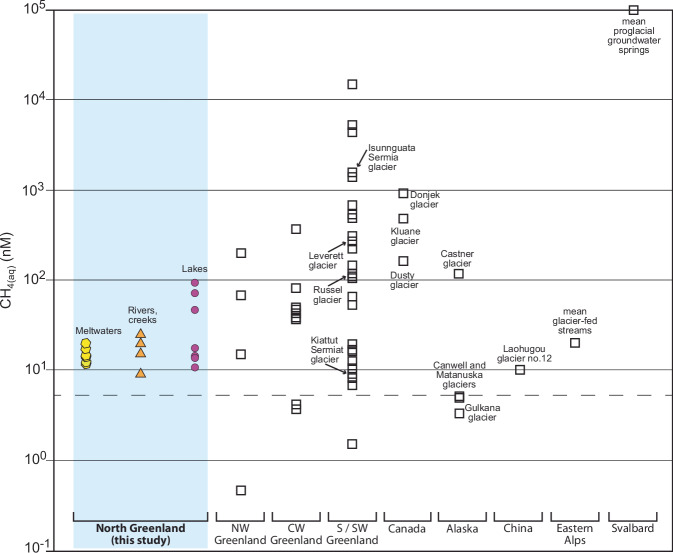
Methane concentrations in meltwaters, periglacial lakes, rivers, and creeks. The diagram shows meltwater (yellow hexagons; avg. 15.2 nM, max. 19.7 nM, min. 11.8 nM), rivers and creeks (orange triangles; avg. 17.1 nM, max. 24.7 nM, min. 9 nM), lakes (purple circles; avg. 38.2 nM, max. 93.6 nM, min. 10.8 nM) data for North Greenland (blue area) but also methane concentrations for south and southwest (avg. 1071 nM, max. 14,026 nM, min. 1.4 nM), central west (avg. 70 nM, max. 347 nM, min. 9 nM), and northwest Greenland meltwaters (avg. 65 nM, max. 184 nM, min. 0.4 nM), and proglacial waters in Canada, Alaska, China, Eastern Alps, and Svalbard (empty squares) for comparison. Notably, the lowest methane concentrations are associated with cold-based glaciers in the southern (e.g., Kiattuut Sermiat), northern (this study), and northwestern Greenland Ice Sheet, whereas the highest concentrations occur in the southwestern sector (e.g., Isunnguata Sermia), where glacier beds are predominantly thawed^31^. Comparison data were obtained from Christiansen et al.^8^, Pain et al.^5^, Lamarche-Gagnon et al.^3^, Sapper et al.^22^, Konya et al.^23^, Du et al.^24^, Ragnoli et al.^48^, Kleber et al.^14^, and Hatton et al.^7^. Selected glaciers mentioned in the text are shown. The dashed line indicates the atmospheric equilibrium concentration of methane (4.9 nM)^7^.

Dissolved gases in meltwaters and in periglacial creeks, rivers, and lakes consist primarily of methane and carbon dioxide; no heavier hydrocarbons were detected. Methane concentrations in meltwaters range from 12–20 nM, similar to those in periglacial rivers and creeks (9–24.7 nM). Periglacial lakes exhibit a broader range and higher values (10.8–93.5 nM; Fig. 2; Supplementary Table 1). All analysed waters are supersaturated with methane—by up to a factor of 20—relative to atmospheric equilibrium (4.9 nM)^7^. Despite this supersaturation, the measured methane concentrations are 1–3 orders of magnitude lower than those reported for meltwaters in southwestern Greenland (e.g., Russell Glacier, 110 nM; Leverett Glacier, 270 nM; Isunnguata Sermia, 1,575 nM)^3,5,7,8^. However, they are similar to some glaciers in the Northwest Greenland^7^ and exceed concentrations measured in southern Greenland, where values are near atmospheric equilibrium (e.g., Kiattut Sermiat, 9 nM)^5,7^. Concentrations are also lower than those reported for several glacial meltwater systems elsewhere, such as in Canada (136–899 nM)^22^, but fall within the range observed in Alaska (2.8–120 nM)^23^ and China (9–16 nM; Fig. 2)^24^.

The relatively low methane concentrations observed in our study area are unexpected, given that the northern margin of the GrIS overlies an actively degassing petroleum system. In analogous geological settings such as Svalbard, methane concentrations in proglacial waters fed by groundwater springs are several orders of magnitude higher (up to 10^6 ^nM)^14^. We did not observe such features in the study area. The concentrations measured here are also lower than those reported from regions where glaciers rest directly on crystalline bedrock, including southwestern Greenland^3^. The processes responsible for the low methane concentrations potentially include: (1) specific characteristics of the underlying petroleum system, such as low gas content or extensive prior degassing; (2) the spatial extent of the petroleum system beneath the northern GrIS^25^ (Supplementary Fig. 3), (3) low rates of subglacial microbial methanogenesis, potentially due to limited availability of organic substrates, (4) the absence of long-term microbial methanogenesis within bedrock fracture networks^26^, (5) methane oxidation within the subglacial environment^3^, (6) early hydrocarbon migration during the Devonian, combined with effective degassing associated with major basin uplift during the Late Devonian to Early Carboniferous (370–350 Ma)^27,28^, (7) possible shorter water residence time in the subglacial environment of northern Greenland relative to southern GrIS basins^29,30^, (8) the existence of a thick permafrost acting as a seal for deep gas migration^11^, and (9) the existence of cold-based glacier patches in North Greenland that limit basal meltwater production^31^. Cold-based glaciers are also present in northwest and south Greenland^31^, where methane concentrations are similarly low^7^. The low methane concentrations measured in North Greenland can also be attributed to sampling at the end of the melt season, which in this region typically occurs in September^32^. Consistent with this seasonal effect, late‑season meltwater measurements from the southwestern GrIS show an approximately ten‑fold decrease in methane concentrations relative to peak melt‑season values, reaching levels comparable to those observed in this study (ca. 18 nM)^3^. Despite lower concentrations, meltwaters in northern Greenland remain consistently supersaturated with methane and thus act as a net atmospheric source.

### Stable carbon isotopes

**Fig. 3:**
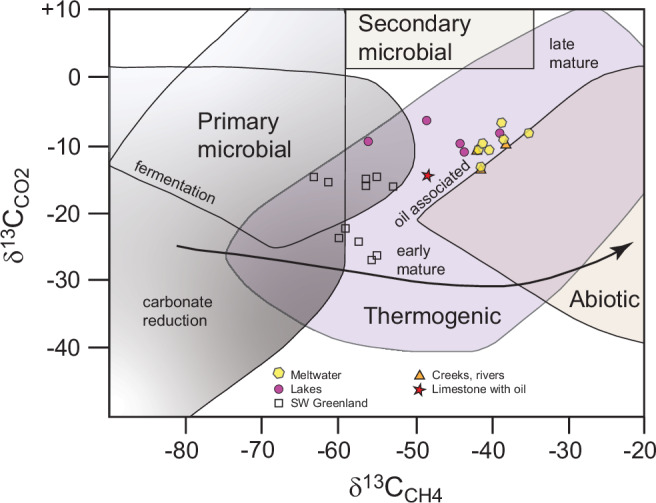
Genetic diagram for methane. Carbon stable isotopes of methane (δ^13^C_CH4_) and carbon dioxide (δ^13^C_CO2_) in waters sampled in North Greenland: meltwater (yellow hexagons), rivers and creeks (orange triangles), and lakes (purple circles). Subglacial meltwaters from southwestern Greenland^3^ (empty squares) and degassed limestone with oil inclusions of the Hauge Bjerge Formation, Franklinian Basin (red star) were plotted for comparison. The sample from this study, located within the “Primary microbial” field, originates from a lake that shows no clear connection to glacial runoff (site 8LAN). The arrow indicates the methane oxidation trend. Diagram fields from Milkov and Etiope^36^.

Stable carbon isotopes of methane and associated carbon dioxide were used to elucidate the origin of methane. Meltwaters exhibit δ^13^C_CH4_ values ranging from − 41.9‰ to −35.1‰ and δ^13^C_CO2_ values between −13.6‰ and −7‰ (Supplementary Table 1). These values reflect methane that is markedly enriched in ¹³C relative to that dissolved in glacial meltwaters elsewhere across the GrIS, where values are typically < −50‰^3,5,33,34^. Periglacial rivers and creeks display comparable isotopic compositions, with δ^13^C_CH4_ values from −42.1‰ to −38.2‰ and δ^13^C_CO2_ values from −13.4‰ to −9.6‰. In contrast, periglacial lakes show a broader range, particularly for methane, with δ^13^C_CH4_ values spanning −58.6‰ to −38.8‰ and δ^13^C_CO2_ values from −11.3‰ to −6.7‰ (Supplementary Table 1). For comparison, we also analysed gas trapped in a bitumen-bearing limestone sample from Polaris Foreland (Hauge Bjerge Formation; Fig. 1 and Supplementary Figs. 1 and 2). This gas contains heavier hydrocarbons (ethane, propane, butane, and pentane) with a methane-to-heavier‑hydrocarbon ratio of approximately seven and exhibits δ^13^C_CH4_ of −48.6‰ and δ^13^C_CO2_ of −14.2‰. These signatures clearly indicate a thermogenic, oil-associated origin (Fig. 3). Despite the absence of measurable heavier hydrocarbons in meltwater and periglacial stream samples, their isotopic signatures indicate a predominantly thermogenic methane source, and/or potential influence of oxidation processes. (Fig. 3). However, several additional lines of evidence support a thermogenic origin: (1) the combined δ^13^C_CH4_ and δ^13^C_CO2_ values plot within the field characteristics of oil-associated thermogenic methane; (2) isotopic signatures resulting from oxidation of microbial methane typically exhibit much wider δ^13^C_CH4_ and δ^13^C_CO2_ ranges^22,35–37^, (3) active degassing of underlying petroleum systems is documented^11^, (4) the close match between the isotopic composition of methane and carbon dioxide in meltwaters and in the associated bitumen-rich bedrock, and (5) shorter water residence time in the subglacial environment^29,30^, which limit the development of strongly reducing conditions required for methanogenesis and interaction times in cold subglacial environments where metabolic rates are inherently slow^1,38,39^. The δ^13^C_CH4_ and δ^13^C_CO2_ values from periglacial lakes similarly point to a predominantly thermogenic, oil‑associated source, except in lakes that are not clearly connected to glacial runoff. These isolated lakes show distinctly microbial signatures^36^, with δ^13^C_CH4_ < −56‰ (i.e., sites 5LAN and 8LAN; Fig. 1; Supplementary Table 1).

### Implications

The thermogenic origin of methane beneath the North GrIS contrasts sharply with observations elsewhere in Greenland, where high dissolved methane concentrations are typically attributed to microbial methanogenesis within subglacial sediments^3,4,7,34^. The comparatively low concentrations observed in northern Greenland demonstrate that methane storage beneath ice sheets cannot be inferred from bedrock type alone (e.g., degassing sedimentary units), and that additional factors such as sampling location and timing relative to the melt season may also exert a strong influence. In addition, the presence or absence of organic‑rich sediments plays a critical role in sustaining microbial methanogenesis and generating elevated methane concentrations in meltwaters—sometimes exceeding 3 × 10⁴ nM⁴— even where glaciers overlie crystalline bedrock^1,4,6,7,40^. The expression of this microbial potential is modulated by hydrological residence times, which limit microbial processing under cold subglacial conditions, as well as by deglaciation history and permafrost evolution, which regulate the quantity and reactivity of organic carbon. Meltwaters from cold-based glaciers in the North, Northwest, and South Greenland^31^ contain, in general, lower methane concentrations than the ones in Southwest and West Greenland^7^, which have warmer, thawed beds (Supplementary Fig. 3)^31^. In addition, large permafrost‑affected areas of the pan‑Arctic shelf, for instance, contain organic carbon that is poorly reactive, thereby limiting microbial methanogenesis and increasing the relative contribution of deeper methane sources at the surface^41^. This framework is consistent with our observations, in which methane is predominantly derived from geological sources independently of subglacial biogeochemical processes. Further permafrost aggradation following glacier retreat may, however, form effective barriers to methane migration from depth^11^, thereby reducing its expression at the surface.

Our results identify a previously under-recognised methane source beneath the GrIS—degassing from petroleum‑bearing formations—and highlight the need for geological methane contributions to be incorporated into methane emission budgets. The magnitude and persistence of this geological source warrant further investigation. The extent to which these findings can be extrapolated across the northern sector of the GrIS remains poorly constrained. However, our observations from major drainage systems, including Peterman and C.H. Ostenfeld glaciers, together with the southward extent of petroleum systems within the Franklinian Basin (Supplementary Fig. 3), suggest that thermogenic methane may be distributed over a broad area beneath the northern GrIS. The western limit of thermogenic influence aligns with the margin of the Franklinian Basin, beyond which methane in the northwestern GrIS is primarily microbial (Supplementary Fig. 3)^7^. We further hypothesise that continued GrIS retreat could enhance petroleum‑system degassing by reactivating underlying tectonic structures^12,13^, potentially increasing methane delivery to subglacial drainage networks and the atmosphere. In addition, our findings suggest that higher methane concentrations are more likely beneath the GrIS in regions hosting younger petroleum systems, such as the Nuussuaq Basin with Cretaceous and Palaeogene source rocks in West Greenland^42^.

Our measurements of methane in meltwaters and periglacial rivers, creeks, and lakes across North Greenland expand the limited dataset of greenhouse‑gas observations from the High Arctic. These measurements reveal pronounced spatial variability in both the concentration and isotopic composition of subglacial methane associated with the GrIS. By providing constraints from North Greenland, this dataset improves the empirical basis for estimating methane emissions from the rapidly retreating ice sheet. These observations also offer essential benchmarks for evaluating and refining existing and future modelling efforts^2,43^, and underscore the need to incorporate potential geological methane sources when assessing the contributions of large ice masses to Arctic and global carbon budgets.

## Methods

### Methane concentration

Concentrations of methane dissolved in water were quantified at Linnaeus University using a headspace equilibration method^44^. A 315‑mL glass vial was completely filled with sample water and amended with 3 mL of the bactericide benzalkonium chloride. The vials were sealed with rubber stoppers and aluminium crimps, and stored upside down at 4 °C. A 60‑mL headspace was created by removing 60 mL of water while simultaneously injecting an equal volume of nitrogen gas. A 0.5‑mL aliquot of the resulting headspace gas was injected into a ThermoFisher TRACE 1310 gas chromatograph (GC) equipped with a PoraPLOT‑Q column and flame‑ionisation (FID) and thermal‑conductivity (TCD) detectors. The GC oven was held at 80 °C for 8 min and then at 150 °C for 5 min; FID and TCD temperatures were maintained at 250 °C. Helium served as the carrier gas at a constant flow of 100 mL min⁻¹. Methane and carbon dioxide concentrations were quantified using calibration curves generated from three standard gas mixtures (CH₄: 10, 100, 1000 ppm; CO₂: 200, 400, 4000 ppm) diluted in helium.

The molar quantity of methane n in the headspace was calculated using the ideal gas law:

1 $${{{\rm{n}}}}_{{{\rm{CH}}}4}=({P}_{{\rm{CH}}4{HS}}\,{{\rm{x}}}{V}_{{\rm{HS}}}{{\rm{x}}}P)/({{\rm{R}}}\; {{\rm{x}}}\; {{\rm{T}}})$$

where P_CH4 HS_ is the measured methane mixing ratio in the headspace (ppmv), *V*_HS_ is headspace volume, *P* is pressure, *R* is the ideal gas constant, and *T* is temperature. The molar quantity of dissolved methane was calculated with the same equation above and the Bunsen coefficient for methane as a function of salinity and temperature, which was obtained with the equation described in Yamamoto et al.^45^:

2 $${{\rm{Ln}}}{{\rm{\beta }}}={{{\rm{A}}}}_{1}+{{{\rm{A}}}}_{2}\left(\frac{100}{{{\rm{T}}}}\right)+{{{\rm{A}}}}_{3}{{\rm{x}}}{\mathrm{ln}}({{\rm{T}}}/100)+{{\rm{S}}}({{{\rm{B}}}}_{1}+{{{\rm{B}}}}_{2}({{\rm{T}}}/100)+{{{\rm{B}}}}_{3}{({{\rm{T}}}/100)}^{2})$$

where *A*_1_, *A*_2_, *A*_3_ and *B*_1_, *B*_2_, and *B*_3_ are constants^45^, *T* is temperature, and *S* is salinity. Methane concentration was then calculated according to the formula:

3 $$[{{{\rm{CH}}}}_{4}]={{{\rm{n}}}}_{{{\rm{CH}}}4}/{{{\rm{V}}}}_{{{\rm{water}}}}$$

### Stable carbon isotopes

Carbon‑isotopic compositions of methane (δ^13^C_CH4_) were determined using the 315‑mL vials prepared as described above, from which a 60‑mL headspace was created. The entire headspace volume was transferred under a helium stream to a concentration unit comprising one chemical trap, three cryogenic traps, and a combustion oven. The resulting CO₂ was analysed using a Thermo Fisher GC‑IsoLink system coupled to a Delta V Plus isotope‑ratio mass spectrometer (GC‑IRMS). Isotopic ratios are reported in δ notation relative to the Vienna Pee Dee Belemnite (VPDB) standard, and samples were compared to a reference gas with δ¹³C = −35.07‰. The δD_CH4_ measurements could not be conducted because methane concentrations were extremely low and sample volumes were prioritised for δ¹³C_CH4_ analyses.

The concentration and carbon‑isotopic composition of carbon dioxide dissolved in water (δ^13^C_CO2_) were determined using duplicate 315 mL vials with 20 mL headspace volume prepared as described above for methane. Concentration was determined by injecting 0.5mL of the headspace gas into the GC, while the carbon isotope was determined by injecting the same volume of gas into the GC-IRMS under the same conditions used for methane analyses. Hydrocarbons and CO₂ trapped in a bitumen‑bearing rock sample were extracted by crushing the rock into fragments <1 cm and allowing gases to degas within gas‑tight jars (isojars) commonly used for borehole cuttings. A 0.5 mL aliquot of the released gas was injected into both the GC and GC‑IRMS to determine molecular and isotopic compositions using the same analytical conditions described above.

## Supplementary information

Supplementary Information

Transparent Peer Review file

## Data Availability

The authors declare that all data supporting the findings of this study are available within the paper and its supplementary information files.
