# Supplementary material for: Thermogenic methane beneath the North Greenland Ice Sheet revealed by isotopic and geological evidence
Source: Nat Commun. 2026 Jul 24;17:7265. doi: 10.1038/s41467-026-75951-4 (PMC13400647; doi:10.1038/s41467-026-75951-4)
Supplement: Supplementary file 1 — Supplementary Information [file 41467_2026_75951_MOESM1_ESM.pdf]

SUPPLEMENTARY INFORMATION

Thermogenic methane beneath the North Greenland Ice Sheet revealed by isotopic and geological evidence.

Ketzer, M.*[1], Jakobsson, M.[2], Faehnrich, K.[3,4], Akhoudas, C.[5], Prytherch, J.[4], Chang, C.[1], Yu, C.X.[1], Sundberg, M.[1], Drake, H.[1], Lattaud, J.[2,6], Hong, W-L.[2], O'Regan, M.[2], Kirchner, N.[7], and Stranne, C[2].

* Corresponding author (marcelo.ketzer@lnu.se)

1. Department of Biology and Environmental Science, Centre for the Environment (CENWIN) Linnaeus University, Kalmar, Sweden.
2. Department of Geological Sciences, Stockholm University, Stockholm, Sweden.
3. College of Sciences, Adelaide University, Adelaide, Australia.
4. Department of Earth Sciences, Uppsala University, Uppsala, Sweden.
5. Bolin Centre for Climate Research, Stockholm University, Stockholm, Sweden.
6. Department of Environmental Science, Stockholm University, Stockholm, Sweden.
7. Department of Physical Geography, Tarfala Research Station, Stockholm University, Stockholm, Sweden.

SUPPLEMENTARY NOTES

Bedrock and sediment cover in the study area

The bedrock consists of Neoproterozoic to Palaeozoic sedimentary successions of the Franklinian Basin, deposited in shelf and deep-water environments (Supplementary Figure 2). These units host petroleum systems with distinct source rocks, including (i) Cambrian carbonates and (ii) Silurian shales, with thermal maturity increasing northward from immature to post-mature and locally metamorphic conditions[1,2]. The limestone sampled for comparative isotopic analyses belongs to the Hauge Bjerge Formation (Aeronian–Telychian, ~441–433 Ma) of the Peary Land Group.

Onshore sediments directly overlying the bedrock consist predominantly of coarse-grained material (gravel), occurring in subglacial settings as well as in lateral and frontal moraines. Finer-grained sediments are mainly associated with the proglacial environment, where glaciofluvial deposits include sand in addition to gravel, and lacustrine deposits are dominated by mud. Lacustrine deposits, in particular, have been cored during previous expeditions and consist primarily of light- to dark-grey silty clay with organic-rich intervals[3]. Sediment thickness locally exceeds 8 m. However, these sediments are spatially limited and of post-glacial (Holocene) age[3] and therefore do not contribute substantially to methane generation beneath the Greenland Ice Sheet (GrIS). The absence of older sediments is also observed in sediment cores from Sherard Osborn Fjord, where Holocene sediments rest directly on bedrock[4]. These observations suggest that thick, organic-rich sediments capable of contributing to methane generation beneath the GrIS in the study area are unlikely, although their presence remains uncertain.

SUPPLEMENTARY FIGURES

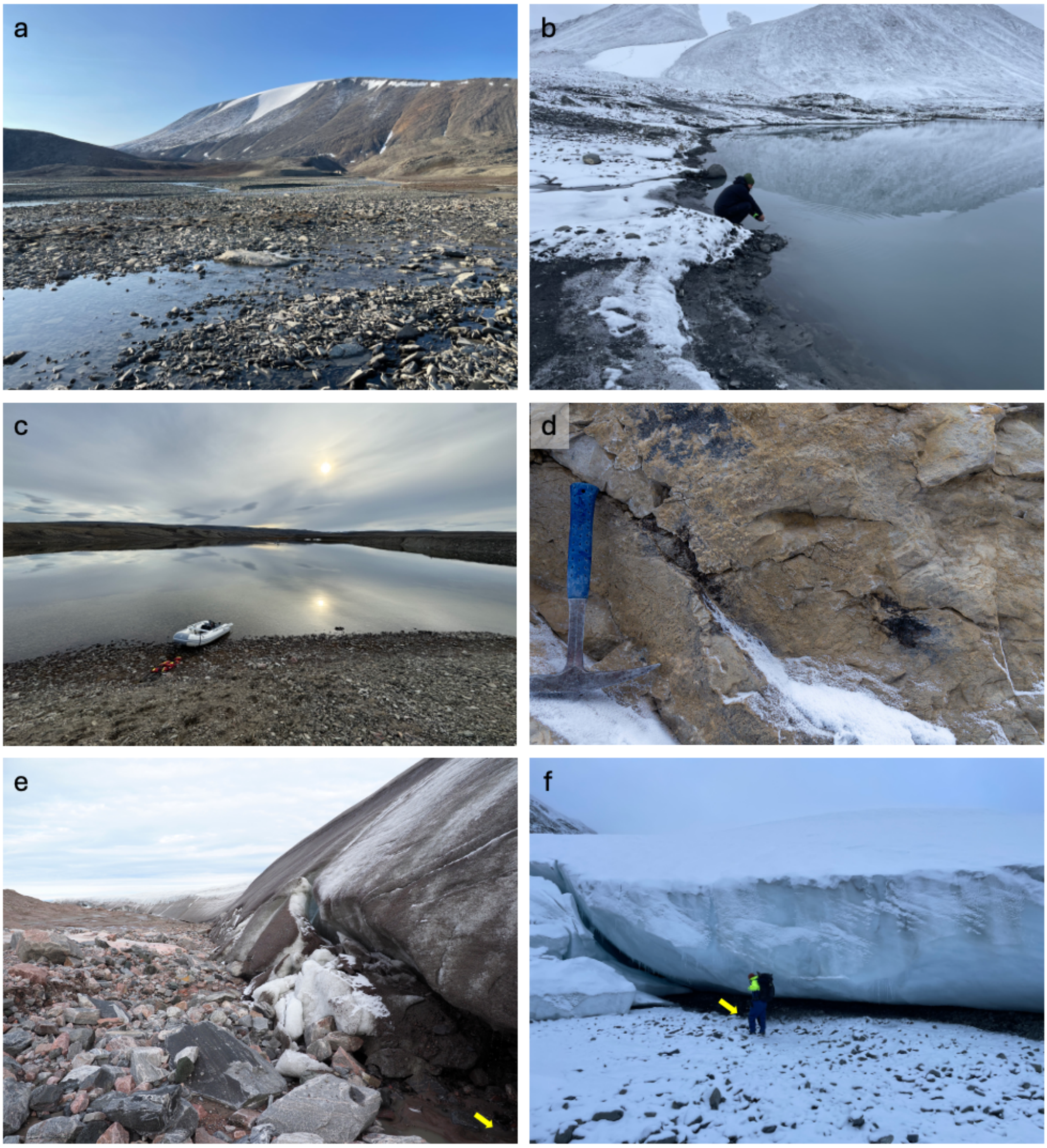

Supplementary Figure 1 – Photographs showing examples of sampling points in North Greenland. **a** Periglacial river in Nares Land (site 02_LAN). **b** Periglacial lake in Freuchen Land (site 17_LAN) with clear connection to glacial runoff. **c** Lake in Wulff Land (site 08_LAN) with no clear connection to glacial runoff. **d** Outcrop of limestone with oil of the Hauge Bjerge Formation in Polaris Foreland (site 25_LAN). **e** Meltwater site from C.H. Ostenfeld glacier in Victoria Fjord (site 07_LAN). **f** Meltwater site from the glacier RGI2000-v7.0-G-05-18484[5] in Nares Land (site 13_LAN).

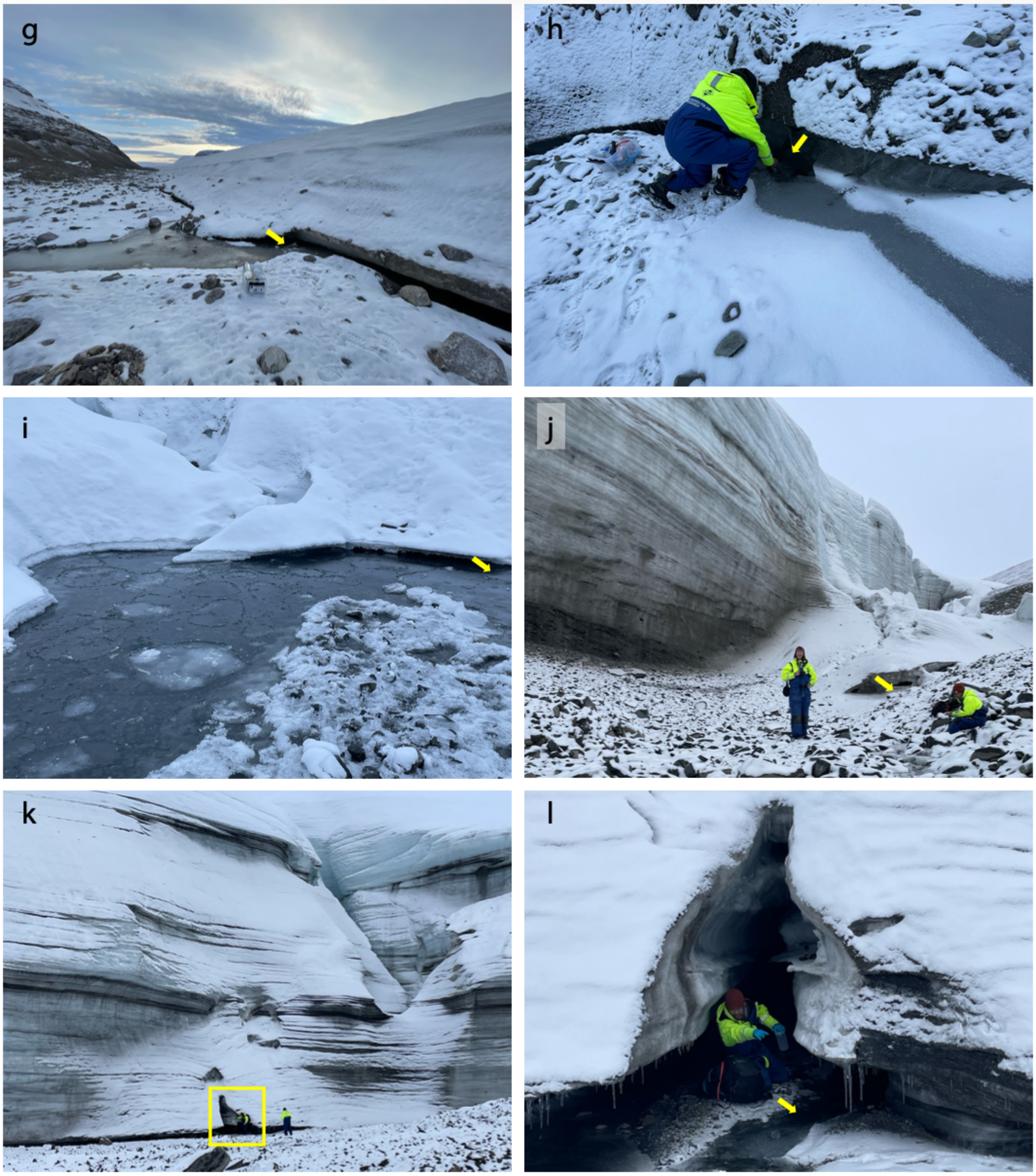

Supplementary Figure 1 cont. g Meltwater site from Petermann glacier in Peterman Fjord (site 26LAN_1). **h** Meltwater site from a glacier near Victoria Fjord in Nares Land (site 16LAN). **i** Meltwater site from a glacier near Victoria Fjord in Nares Land (site 14LAN). **j** Meltwater site from glacier near Nordensskjöld Fjord in Freuchen Land (site 19LAN). **k** Glacier near Nordensskjöld Fjord in Freuchen Land and location of the sampling point 18LAN (yellow square). **l** Meltwater site from glacier near Nordensskjöld Fjord in Freuchen Land (site 18LAN). Arrows on the photographs mark the approximate location of samples. See Figure 1, Supplementary Figure 2, and Supplementary Table 1 for location.

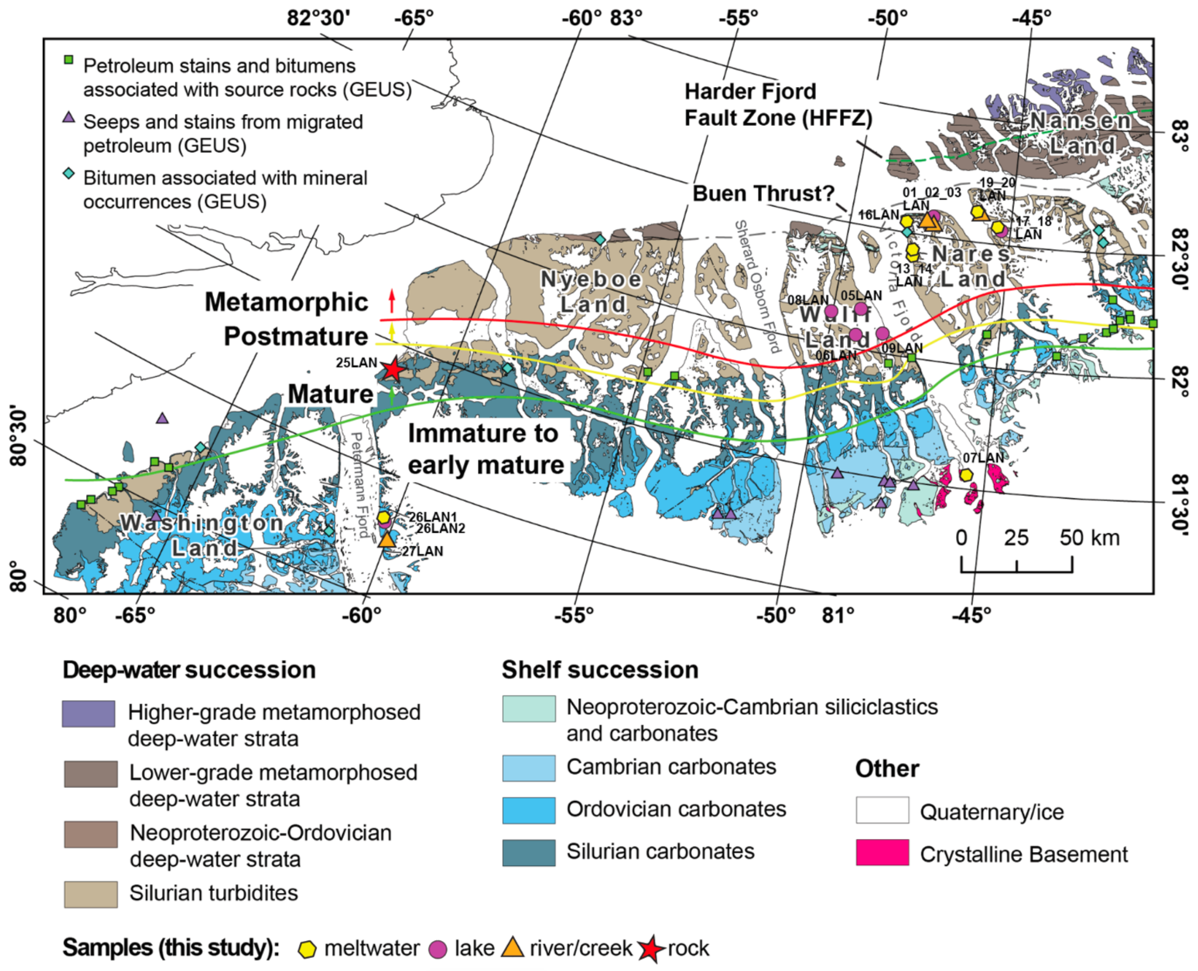

Supplementary Figure 2 - Generalised geological map of the area of study with locations of rock outcrops with oil stains and seeps using data from the Geological Survey of Denmark and Greenland (GEUS) and the Mineral Resources Authority (MMR), Naalakkersuisut – Government of Greenland[6,7]. The bedrock in the study area is composed of Neoproterozoic to Palaeozoic units of the Franklinian Basin deposited in shelf and deep-water environments. These units host petroleum systems with distinct source rocks: (i) Cambrian carbonates and (ii) Silurian shales, with thermal maturity increasing from immature, to postmature and metamorphic towards north[1,2]. Lines indicate thermal maturity, increasing towards the north, from Christiansen et al.[8]. Geological divisions modified after Henriksen et al.[9] and Hopper and Ineson[10]. Data reproduced/adapted from Geological Survey of Denmark and Greenland (GEUS), Geological Map of Greenland, 1:500.000 (2024) and Geological Survey of Denmark and Greenland (GEUS) and the Mineral Resources Authority (MMR), Inventory of Onshore Petroleum Seeps and Stains in Greenland. (2024), licensed under Creative Commons Attribution 4.0 International (CC BY 4.0; https://creativecommons.org/licenses/by/4.0/). Changes were made to the original material.

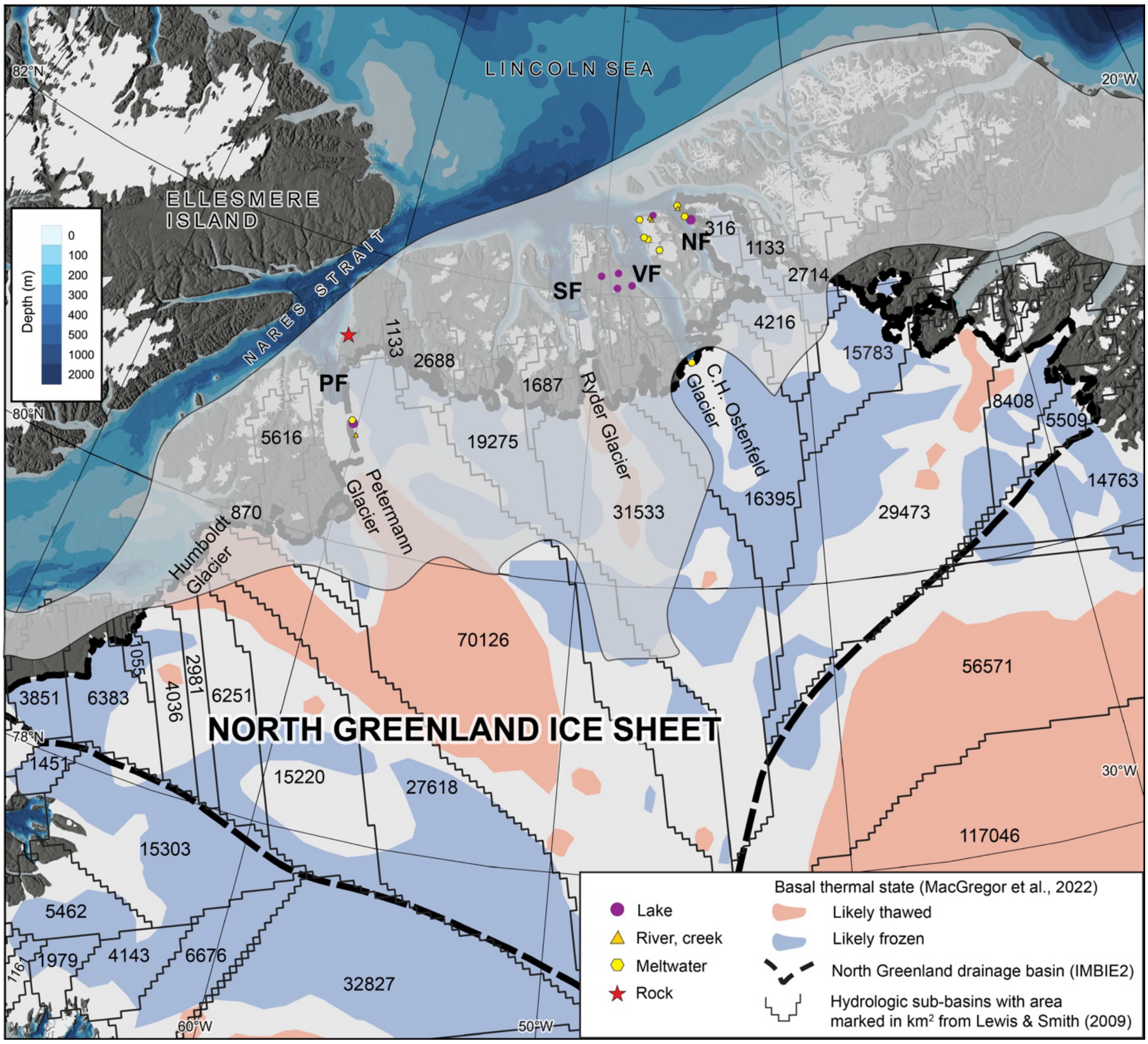

Supplementary Figure 3 – Map showing the present-day extent of the Franklinian Basin beneath the North Greenland Ice Sheet (grey area) and the thermal state of the Greenland Ice Sheet bed: frozen (blue areas) and thawed (orange areas). Abbreviations indicate the names of the fjords: Petermann Fjord (PF), Sherard Osborn Fjord (SF), Viktoria Fjord (VF), and Nordenskjöld Fjord (NF). Data from MacGregor et al.[11] and Henriksen et al.[9]. The map also shows the North Greenland Ice sheet (dashed line) with drainage areas of different catchments (numbers, in km^2) and the sampling points for melt water (yellow hexagons) and rock with bitumen (red star). Bathymetric data are from the International Bathymetric Chart of the Arctic Ocean (IBCAO) Version 5.0[12], which incorporates BedMachine Version 5 data for the inner-fjord bathymetry and subglacial topography[13]. The bathymetry of Victoria Fjord and Nordenskjöld Fjord has been updated using data collected during the GEOEO 2024 expedition[14]. Hydrologic sub-basins of the Greenland Ice Sheet are from Lewis and Smith[15], while the thermal basal conditions of the Greenland Ice Sheet (frozen or thawed) are a simplified, generalized representation adapted from MacGregor, et al. GBaTSv2: a revised synthesis of the likely basal thermal state of the Greenland Ice Sheet. The Cryosphere 16, 3033-3049 (2022). https://doi.org/10.5194/tc-16-3033-2022 and licensed under a CC BY license: https://creativecommons.org/licenses/by/4.0/ [16]. The extent of the Franklinian Basin is overlaid using the geological information shown in Supplementary Figure 2.

SUPPLEMENTARY TABLE

Supplementary Table 1 - Coordinates of sampling sites, sampling dates, methane and carbon dioxide concentrations and isotopic compositions in water samples.

Station	Latitude	Longitude	sampling date	CH_4 nM	$\delta^{13}C$ CH_4 ‰	CO_2 uM**	$\delta^{13}C$ CO_2 ‰
creeks, rivers							
02LAN	82.56335	-47.59613	2024-08-21	14.89	-42.148	23.52	-10,706
03LAN	82.56703	-47.5915	2024-08-21	9.04		23.37	-11,514
20LAN	82.61205	-46.02867	2024-08-30	19.66	-38.191	32.12	-9,624
27LAN	80.74338	-59.99279	2024-09-13	24.67	-41.62	25.53	-13,356
meltwater							
13LAN	82.41628	-47.93	2024-08-30	19.74	-41.539	11.9	-13,552
14LAN	82.41628	-47.93	2024-08-30	17.08	-41.063	18.97	-10,951
16LAN	82.53856	-48.243861	2024-08-30	11.8	-38.337	17.56	-9,97
07LAN	81.557894	-45.462784	2024-08-21	12.31	-41.976	12.96	-9,507
18LAN	82.57102	-45.42372	2024-08-30	13.71	-38.692	14.08	-6,98
19LAN	82.61205	-46.02867	2024-08-30	17.2	-40.136	19.19	-10,898
26LAN_1	80.81487	-60.28006	2024-09-13	14.44	-35.067	40.27	-8,621
lakes							
01LAN	82.56865	-47.55682	2024-08-21	10.77		15.7	-10,248
05LAN*	82.16	-49.163	2024-08-22	93.55	-58.632		
06LAN	82.054	-49.163	2024-08-22	14.24	-48.489	38.85	-6,773
08LAN*	82.12562	-50.00187	2024-08-27	70.83	-56.189	37.38	-9,714
09LAN	82.07493	-48.42754	2024-08-28	13.62	-43.513	36.39	-11,347
17LAN	82.57102	-45.42372	2024-08-30	46.67	-43.953	14.12	-10,212
26LAN_2	80.81734	-60.28022	2024-09-13	17.48	-38.814	27.5	-8,566
limestone with oil							
25_LAN	81.39769	-61.46068	2024-09-10		-48.6		-14.152

* Lakes without clear connection to glacial runoff.

** CO_2 concentrations were calculated using the ideal gas law and Henry's constant[17].

SUPPLEMENTARY REFERENCES:

1 Stemmerik, L., Sønderholm, M., Bojesen-Koefoe, J.A. Palaeo-oil field in a Silurian carbonate buildup,Wulff Land, North Greenland: project 'Resources of the sedimentary basins of North and East Greenland'. *Geology of Greenland Survey Bulletin* **176**, 24-28 (1997). https://doi.org/10.34194/ggub.v176.5056

2 Christiansen, F. G., Bojesen-Koefoed, J.A. Inventory of onshore petroleum seeps and stains in Greenland: a web-based GIS model. *Geological Survey of Denmark and Greenland Bulletin* **47** (2021). https://doi.org/10.34194/geusb.v47.6519

3 Jakobsson, M., Mayer, L., Farrel, J. SWEDARTIC Ryder 2019 Expedition Final Report. 456 (Luleå, 2019).

4 O'Regan, M., Cronin, T.M., Reily, B., Alstrup, A.K.O., Gemery, L., Golub, A., Mayer, L.A., Morlighem, M., Moros, M., Munk, O.L., Nilsson, J., Pearce, C., Detlef, H., Stranne, C., Vermassen, F., West, G., Jakobsson, M. The Holocene dynamics of Ryder Glacier and ice tongue in north Greenland. *The Crysosphere* **15**, 4073-4097 (2021). https://doi.org/10.5194/tc-15-4073-2021

5 Consortium, R. G. I. Randolph Glacier Inventory - A Dataset of Global Glacier Outlines, Version 7.0. **7** (2023). https://doi.org/doi.org/10.5067/f6jmovy5navz

6 Geological Survey of Denmark and Greenland (GEUS) and the Mineral Resources Authority (MMR), N. G. o. G. Inventory of Onshore Petroleum Seeps and Stains in Greenland. (2024).

7 (GEUS), G. S. o. D. a. G. Geological Map of Greenland, 1:500.000. (2024).

8 Christiansen, F. G., Koch, C.J.W., Nøhr-Hansen, H., Stouge, S., Thomsen, E., Østfeldt, P. in *Petroleum Geology of North Greenland* Vol. 158 (ed F.G. Christiansen) Ch. 6, 40-60 (Geological Survey of Greenland, 1989).

9 Henriksen. N. Higgins, A. K., Kalsbeek, F., Pulvertaft, C.R. Greenland from Archaean to Quaternary. *Geological Survey of Denmark and Greenland Bulletin* **18**, 126 (2009). https://doi.org/10.34194/ggub.v185.5197

10 Hopper, J. R., Ineson, J.R. in *Sedimentary Successions of the Arctic Region and their Hydrocarbon Prospectivity* Vol. 57 *Memoir* (ed S. S. Drachev, Brekke, H., Henriksen, E. and Moore, T.) (Geological Society of London, 2022).

11 MacGregor, J. A., Colgan, W.T., Paxman, G.J.G., Tinto, K.J., Csathó, B., Darbyshire, F.A., Fahnestock, M.A., Kokfeldt, T.F., MacKie, E.J., Morlighem, M., Sergienko, O.V. Geologic Provinces Beneath the Greenland Ice Sheet Constrained by Geophysical Data Synthesis. *Geophysical Research Letters* **51**, e2023GL107357 (2024). https://doi.org/10.1029/2023GL107357

12 Jakobsson, M., Mohammad, R., Karlsson, M., Salas-Romero, S., Vacek, F., Heinze, F., Bringensparr, C., Castro, C.F., Johnson, P., Kinney, J., Cardigos, S., Bogonko, M., Accettella, D., Amblas, D., An, L., Bohan, A., Brandt, A., Bünz, S., Canals, M., Casamor, J.L., Coakley, B., Cornish, N., Danielson, S., Demarte, M., Di Franco, D., Dickson, M.-L., Dorschel, B., Dowdeswell, J.A., Dreutter, S., Fremand, A.C., Hall, J.K., Hally, B., Holland, D., Hong, J.K., Ivaldi, R., Knutz, P.C., Krawczyk, D.W., Kristofferson, Y., Lastras, G., Leck, C., Lucchi, R.G., Masetti, G., Morlighem, M., Muchowski, J., Nielsen, T., Noormets, R., Plaza-Faverola, A., Prescott, M.M., Purser, A., Rasmussen, T.L., Rebesco, M., Rignot, E., Rysgaard, S., Silyakova, A., Snoeijs-Leijonmalm, P., Sørensen, A., Straneo, F., Sutherland, D.A., Tate, A.J., Travaglini, P., Trenholm, N., van Wijk, E., Wallace, L., Willis, J.K., Wood, M., Zimmermann, M., Zinglersen, K.B., Mayer, L. The International

Bathymetric Chart of the Arctic Ocean Version 5.0. *Scientific Data* **11** (2024). https://doi.org/10.1038/s41597-024-04278-w

13 Morlighem, M. (2023).

14 Jakobsson, M., Kirchner, N., Nilsson, J., Stranne, C., Mayer, L., Barnett, J., Holmes, F.A., Calder, B., Deutsch, C., de Boer, A.M., Coxall, H.K., Faehnrich, K., Hong, W-L., Hopper, J.R., Ketzer, M., Noormets, R., O'Regan, M., Ross, N., Sigray, P., Erstorp, E.S., Weidner, E., Wang, Z. Atlantic water access and the break-up of the C.H. Ostenfeld Gletsjer ice tongue, northwest Greenland. *Communications Earth & Environment* **7**, 1-9 (2026). https://doi.org/10.1038/s43247-026-03666-x

15 Lewis, S. M., Smith, L.C. Hydrologic drainage of the Greenland Ice Sheet. *Hydrological Processes* **23**, 2004-2011 (2009). https://doi.org/10.1002/hyp.7343

16 MacGregor, J. A., Chu, W., Colgan, W.T., Fahnestock, M.A., Felikson, D., Karlsson, N.B., Nowicki, S.M.J., Studinger, M. GBaTSv2: a revised synthesis of the likely basal thermal state of the Greenland Ice Sheet. *The Cryosphere* **16**, 3033-3049 (2022). https://doi.org/10.5194/tc-16-3033-2022

17 Weiss, R. F. Carbon dioxide in water and seawater: the solubility of a non-ideal gas. *Marine Chemistry* **2**, 203-215 (1974). https://doi.org/10.1016/0304-4203(74)90015-2
