# Supplementary material for: Thermogenic methane beneath the North Greenland Ice Sheet revealed by isotopic and geological evidence
Source: Nat Commun. 2026 Jul 24;17:7265. doi: 10.1038/s41467-026-75951-4 (PMC13400647; doi:10.1038/s41467-026-75951-4)
Supplement: Supplementary file 2 — Transparent Peer Review file [file 41467_2026_75951_MOESM2_ESM.pdf]

nature portfolio

Peer Review File

Thermogenic methane beneath the North Greenland Ice Sheet revealed by isotopic and geological evidence.

Corresponding Author: Professor Marcelo Ketzer

This file contains all reviewer reports in order by version, followed by all author rebuttals in order by version.

Version 0:

Reviewer comments:

Reviewer #1

(Remarks to the Author)
Review of Nature Communications manuscript NCOMMS-26-019154
It is an excellent and well-written paper. The subject is highly topical and relevant. Knowing that it is from a remote and very difficultly accessible part of the world, the paper and as many background results as possible deserve to be published as soon as possible.
In contrast to many other reviews, I only have a few direct suggestions, and I have only found a few errors or misunderstandings (marked in enclosed files).
I do not see any reason to be an anonymous reviewer; I was lucky enough to participate in field work and drilling in the region in 1984-85 and I was editing a large Bulletin summarizing all information of relevance to petroleum generation and accumulation in the Franklinian Basin (Christiansen ed. 1989). It is very interesting to see new gas data in a completely different context.
I have also been working in many other sedimentary basins in Greenland, and especially the Nuussuaq Basin in West Greenland where gas data may be of similar importance for discussions of biogenic versus thermogenic origin (Christiansen et al. 2020).
Both basins have been covered by ice and with low recent temperatures permafrost is many hundreds of meters thick. Although it is important to discuss what is coming from below the Greenland Ice Sheet, it is for me similarly important to discuss what is coming from below the permafrost, and what was the timing of primary generation, later migration and release during uplift.
In the Franklinian Basin, the source rocks are very old – Cambrian and Silurian – and the organic material marine (no primary gas potential, gas is from later cracking of oil). Generation, migration and accumulation took place very early in history, probably during Devonian compression.
The Nuussuaq Basin is completely different with much younger source rocks – Cretaceous and Palaeogene – and with organic material including lots of terrestrial material that have been generating early gas. Generation was quick after loading of many km of volcanics. Large amounts of oil and gas are most likely still accumulated in the subsurface. Numerous visible seeps and core drilling suggest this.
These differences in timing clearly explain the very low concentration of methane in North Greenland compared to West Greenland. The isotope signatures are rather similar and in the thermogenic ranges. It is a pity that there are no δDC1 values that could give better information on thermal maturity and possible N-S variation.
The discussion in the paper would be even better if it included some comments on the generation and migration history, thermal maturity variation from North to South and uplift history with comments on importance of permafrost and where the gas comes from.

Christiansen, F. G. edit. 1989: Petroleum Geology of North Greenland. Bulletin Grønlands geologiske Undersøgelse 158, 104 pp. https://doi.org/10.34194/bullggu.v158.6700
Christiansen, F. G., Bojesen-Koefoed, J. A., Dam, G., Laier, T. & Salehi, S. 2020: Oil and gas seepage in the Nuussuaq Basin, West Greenland – implications for petroleum exploration. GEUS Bulletin 44, 4567
https://doi.org/10.34194/geusb.v44.4567

Reviewer #2

(Remarks to the Author)
=== General comments

The manuscript by Ketzer et al. presents data on glacial methane from a very understudied and difficult to access area that is northern Greenland. Their results show small dissolved methane concentrations compared to other studies on glacial methane elsewhere in Greenland and other parts of the Arctic, but perhaps more importantly they attribute a thermogenic source of the methane detected based on stable isotope and geological data.

The presence of thermogenic methane in the area is not surprising given the overall geology and prior oil and gas prospective surveys in the area - as pointed out by the authors - but confirming that thermogenic methane is indeed present from glacial sources in northern Greenland is important for future assessments of glacial methane emissions. I agree with the authors that this is much needed data for Greenland especially, and that this type of data can only be collected from very logistically challenging and costly field campaigns. Therefore I think that the data presented is of high relevance to the scientific community.

That being said, I think that some of the data interpretation and conclusions to be oversimplified based on the timing of the study and possible sampling locations which require further clarifications, and I am not convinced that the manuscript as currently written accurately contextualised the findings.

=== Major comments

1. Low methane concentrations and sampling context

Like the authors, I too was surprised about the very low methane concentrations reported for this area of Greenland given the likely thermogenic methane sources, especially when compared to other glaciated areas where thermogenic methane has been reported (e.g. Kleber 2023, 2025). However it is unclear whether these low methane concentrations are representative of concentrations expected to be found beneath the local glacial masses sourcing the sampled meltwaters, or artefacts of sampling locations, timing and seasonality. Samples were collected towards the very end of the melt season in late August/September, at time when I would expect minimal subglacial melt to be routed to the proglacial area. Studies on both Greenland and Svalbard (Lamarche-Gagnon 2019, Kleber 2025) show much lower glacial methane concentrations towards the end vs beginning of the melt-season, mostly due to a combination of increasing dilution of methane sources over the course of the meltseason, extensive flushing of the subglacial environment during the previous months, and meltwater shutdown towards the end of the meltseason. I would expect that for north Greenland, the timing of the study would even have a greater impact than let's say Svalbard given the colder temperatures and shorter melt-season. Perhaps more relevant is comparing the results here with those reported by Lamarche-Gagnon 2019 for late October meltwater samples in SW Greenland, which showed 1 order of magnitude smaller concentrations than during early and peak melt (i.e. ~18 nM vs 200 nM). These late-season SW Greenland concentrations are similar concentrations than those reported by the authors here.

I understand the logistical challenges that come with sampling that area of Greenland, that the authors were likely constrained by the timing of the cruise to collect those samples, that earlier cruises are impractical to impossible in the region due to the presence of sea-ice and also that the many sites sampled by helicopter represent a true effort by the authors. The very high logistical challenges that come with collected these samples are one of the main reasons why these data are very valuable. But to me the reported methane concentrations in this study likely represent lower-concentration "end-members" from late melt-seasons at best.

Moreover, more details regarding the exact sampling locations are needed in addition to only lat/lon coordinates (though those are of course needed). How were those "meltwaters" defined? It is very difficult from satellite images to assess how close to the glacial outlets the meltwater samples were collected and whether these meltwaters were exiting main subglacial water channels/outlets or consisted of lateral/periglacial meltwaters, which would be more diluted. I appreciate including photograph examples of some sampling site as supp material but I would add a photo for each sampling point if possible, especially for the meltwater sites. Even from the provided photographs, it is unclear what constitute the source of the samples waters. E.g. Supp Fig. 1d is labeled as meltwaters but no visible meltwater is present - perhaps maybe under the ice ledge but if so this further highlight my earlier points that late-season samples may not be representative of true subglacial waters.

Further, can the author comment on whether the meltwaters originated from likely cold or warm-based glacial systems? It is my understanding that several of the smaller glacier in the area might be cold-based (unlike Petermann Glacier). I would not expect much methane to be flushed out from cold-based glaciers.

2. CO_2 measurements and stable isotopes, and concomitant characterisation of thermogenic vs oxidised CH_4sources.

I am also a bit concerned by the methods employed to measure dissolved CO_2 and CO_2 carbon stable isotopes; mainly the use of phosphoric acid. Essentially what the authors measured is total DIC as CO_2 and not only dissolved CO_2 concentrations (e.g. Salata et al. 2000). This method can have significant impact on the overall CO_2 concentrations (e.g. Koschorreck et al. 2021), especially for waters with high alkalinity (which if often the case for glacial meltwaters). I believe this can be corrected by using pH and alkalinity but it is not clear from the methods that corrections were mades or that sample pH and alkalinity measured. Unless the authors can demonstrate or reference that 13C-DIC can be used instead of 13C-CO_2 in stable-isotope genetic diagrams to infer methane gas sources.

The above does not completely discredit the results presented and the 13C-Ch4 remain the most important data to attribute a thermogenic methane origin. For what it's worth I still agree with the authors that a thermogenic origin for the methane is the most likely explanation and the authors present other lines of evidence to that regards (e.g. thermogenic methane from degassed limestone samples), but, given that determining whether or not the glacial methane detected is thermogenic is the key finding of the study, evidence needs to be presented as strongly as possible. Especially given that alteration of the stable isotopic signature by microbial oxidation cannot be systematically ruled out.

3. Methane flux calculations

Whilst I commend the authors for their efforts in trying to calculate diffusive fluxes for their sampling sites and comparsison sites, given the very low concentrations reported and snapshot sampling, I believe these fluxes can difficulty be applied in a larger context, are highly speculative given the absence of data on river Q and v, and prevent any useful upscaling calculations and make the comparison with large world rivers not very relevant. I would therefore advise to remove the riverine methane flux comparison with other large rivers. I also have some reservations as to how these riverine fluxes were calculated (see specific comments below). I would simply keep comparisons between other glacial sites and potentially only for concentrations (Fig. 2a).

== specific comments

Ln21: report measured concentration range

Ln25 & 35: I would tone down the language. "challenges" (abstract), "presumes" (Ln 35), etc. The findings are important and indeed previous reports in Western and Southern Greenland only attributed a microbial source for glacial methane, but I don't think any of these studies and follow-ups simply presumed that all glacial methane from Greenland would be strictly microbial, especially given widespread reports of thermogenic methane from glaciated catchments overlaying sedimentary rocks on Svalbard. In fact recent studies specifically allude to the fact that thermogenic methane is likely to be present in northern Greenland (e.g. Wadham et al. 2026). Therefore I would tone down the language a little bit and instead highlight the novelty of the findings as opposed to present those as a challenge to previous work.

Ln 41: Despite the somewhat provocative title by Christiansen 2018, the first direct observations of subglacial methane export in Greenland are from Dieser 2014 and Webster 2015. And technically the first "subglacial observations" could be attributed to deep ice core campaigns that measured elevated methane from basal ice in the 90s. I would therefore re-phrase to "first observations from subglacial meltwaters" and cite Dieser and Webster.

Ln42: "significant atmospheric emissions" This is still up for debate and no studies so far has directly measured or quantified atm emissions of glacial methane. If anything upscaling of diffusive riverine fluxes only amounts to modest emissions globally. I would tone down to something like "potentially significant" or "previously under appreciated"

Ln40-54: I think these are nicely written paragraphs that present and introduce very well the relevance of the study.

Ln54: Would also cite Svalbard work on glacial fracturing (Hodson 2020, Kleber 2023)

Ln60: I would also add relevant to Canadian High Arctic, given the geographical proximity and geological similarities

Fig. 1. Nice map. 1b. I don't see any gas flare (yellow star) highlighted on the map, unlike stated in the legend. Also discrepancies between legend of Fig. S2 re lake vs meltwater sampling sites (e.g. near Peterman fjord). I would make sure all of those are consistent.

Supp. Table 1: Add a column with sampling date. Also CO2 concentrations.

Ln104-116: see previous general comment re low concentrations likely attributed to seasonality (point 6) but perhaps more importantly sampling sites and "true" subglacial samples.

Ln118-127: See previous general comment. I would keep the flux calculations to the Supp Material and avoid comparisons with large river systems.

Ln149: Is the gas-trapped from bitumen containing limestone CH4 stable isotope plotted on Fig. 3? From the figure legend I would guess this is the red star on the figure but the stable istope signature plotted vs the one described in the text seem to differ slightly for 13CO2 - i.e. -8.9 permil in the text vs less than -10 on the figure. This should be corrected, added, or clarified as I agree with the authors that this gas sample is probably one of the best end-member samples they have to compare their field methane samples and attribute to those a thermogenic origin.

Ln143-160: More information is needed to identify the samples/lakes connected or disconnected from glacial runoff. E.g. this could easily be highlighted in SuppTable 1. Same for creeks and rivers earlier in the text - how are those defined and are they completely disconnected hydrologically from glacial influence/runoff (except potentially from groundwaters)? Need more geographical description in the methods/SI and main text. Ln 159 seems to imply that lakes disconnected from glacial

runoff are microbial - I would highlight on the figure.

I also point out that only samples with highest methane concentrations from lakes could be unambiguously attributed to microbial methane. If possible I would add on the figure the expected fractionation trajectory from microbial oxidation. Given the very low methane concentrations of all samples, very difficult to trust at face value the absence of significant microbial oxidation influence.

Ln172 and main conclusion: I am not convinced by the data presented that northern Greenland/visited sites does not overlay larger thermogenic methane reserves, and that the low CH4 concentrations reported by the authors are not simple artefacts of sampling locations and time.

=== methods
Ln240-248: see previous comment regarding CO2 vs DIC measurements and acidification. Unless somehow CO2 concentrations were also derived and measured using the headspace from the CH4 vials and that bensalonium chloride does not significantly impact pH and that CO2 concentrations measure from the headsapce of the methane samples matched CO2 concentrations measured following acidification. Otherwise more details are needed to trust the 13C-CO2 measured following acidification.

ln257: CH4 atm-eq concentration. Given the low dissolved CH4_aq measured by the authors, they should calculate a new CH4 atm-eq concentration based on in situ water temp, salinities, but more importantly background atmospheric concentrations, which in 2024 should be (slightly) higher than in 2015 as per their reference.

Ln258: why 0.22C? is this the average water temperature for all sites? What was the temperature range?

Ln259: why were those stream velocities and slopes used? Was this based on any empirical/GIS data? The stream v seems high for this late period of the melt-season. Can the authors confirm high stream flow for all river/meltwater sites sampled? Doesn't look like it from photos (supp 2)

Ln269: how was water temperature measured?

Ln271: typo -> change Raynolds to Raymond et al.

Ln275-278: I now see the authors are basing their diffusive flux calculations based on LamarcheG-2019 which were calculated for a completely different meltwater-river system in South West Greenland that used in situ measurements and continuous river monitoring. River slope from 1 river-site to another one cannot simply be transposed. Ok to use broad boundary conditions to fork uncertainties but this should still be based on local physical conditions; slope can be quickly obtained from GIS for the areas in question. Or base those on global river surveys - e.g. Raymond 2012-13 model for low slope conditions

But perhaps more importantly and as discussed above, I don't think those flux calculations provide very useful data to contextualise the results.

== References cited in the review

Kleber, G. E. et al. Proglacial methane emissions driven by meltwater and groundwater flushing in a high-Arctic glacial catchment. Biogeosciences 22, 659-674 (2025). https://doi.org:10.5194/bg-22-659-2025

Kleber, G. E. et al. Groundwater springs formed during glacial retreat are a large source of methane in the high Arctic. Nature Geoscience 16, 597-604 (2023). https://doi.org:10.1038/s41561-023-01210-6

Lamarche-Gagnon, G. et al. Greenland melt drives continuous export of methane from the ice-sheet bed. Nature 565, 73-77 (2019). https://doi.org:10.1038/s41586-018-0800-0

Koschorreck, M., Prairie, Y. T., Kim, J. & Marcé, R. Technical note: CO2 is not like CH4 – limits of and corrections to the headspace method to analyse pCO2 in fresh water. Biogeosciences 18, 1619-1627 (2021). https://doi.org:10.5194/bg-18-1619-2021

Hodson, A. J. et al. Sub-permafrost methane seepage from open-system pingos in Svalbard. The Cryosphere 14, 3829-3842 (2020). https://doi.org:10.5194/tc-14-3829-2020

Webster, K. D., White, J. R. & Pratt, L. M. Ground-Level Concentrations of Atmospheric Methane in Southwest Greenland Evaluated Using Open-Path Laser Spectroscopy and Cavity-Enhanced Absorption Spectroscopy. Arctic, Antarctic, and Alpine Research 47, 599-609 (2015). https://doi.org:10.1657/AAAR0014-051

Raymond, P. A. et al. Global carbon dioxide emissions from inland waters. Nature 503, 355-359 (2013). https://doi.org:10.1038/nature12760

Raymond, P. A. et al. Scaling the gas transfer velocity and hydraulic geometry in streams and small rivers. Limnology and Oceanography: Fluids and Environments 2, 41-53 (2012). https://doi.org:10.1215/21573689-1597669

Salata, G. G., Roelke, L. A. & Cifuentes, L. A. A rapid and precise method for measuring stable carbon isotope ratios of dissolved inorganic carbon. Marine Chemistry 69, 153-161 (2000). https://doi.org:https://doi.org/10.1016/S0304-4203(99)00102-4

Wadham, J. L. et al. An Ice Sheet-to-Ocean Analysis of Carbon Stores and Fluxes in Earth's Polar Regions (RECCAP2, Polar Ice Sheets). Global Biogeochemical Cycles 40, e2025GB008677 (2026). https://doi.org:https://doi.org/10.1029/2025GB008677

Reviewer #3

(Remarks to the Author)
General assessment
This manuscript addresses a timely and important topic: methane cycling in Arctic glacial catchments. The combined use of dissolved methane concentrations and stable isotope data in meltwaters is particularly valuable, as such datasets remain sparse in the literature. The study provides important evidence that thermogenic methane constitutes a substantial fraction of the exported methane signal, implying that continued glacier retreat may enhance fluxes of geologically sourced methane to the atmosphere. This finding is highly relevant in the context of glacier recession and is consistent with previous observations of cryosphere-controlled methane release pathways (e.g. Walter Anthony et al., 2012; Lamarche Gagnon et al., 2019).
Overall, the manuscript is clearly written and the conclusions are generally supported by the presented results. However, several aspects related to site representativeness, subglacial and proglacial context, and process interpretation would benefit from clarification. Addressing these points would strengthen the manuscript and improve its relevance for a broad readership. I therefore recommend moderate revision.
Major comments
1. Representativeness of the study site within the Greenland Ice Sheet (GrIS) context
The manuscript would benefit from a clearer positioning of the investigated catchment relative to the Greenland Ice Sheet. It is not fully clear whether the study site is intended to represent a typical GrIS drainage system in northern Greenland, or whether it should instead be considered a glacierised basin adjacent to, but not fully representative of, the ice sheet. Providing additional information on the glaciological setting, including drainage area characteristics and its relation to the GrIS, would help readers better assess the broader applicability of the results. Alternatively, the discussion could more explicitly frame the findings in the context of glacierised basins outside the main ice sheet. Such clarification is particularly important given that subglacial sediment thickness beneath parts of the GrIS can be substantial (e.g. Lamarche Gagnon et al., 2019), whereas sediment properties at the study site are not clearly constrained.
2. Subglacial drainage and water residence time
Key aspects of the local subglacial hydrological setting, such as drainage efficiency and water residence time, are only briefly addressed in the manuscript, despite their central role in controlling methane production and export. A more explicit description, even at a qualitative level, of the expected drainage configuration and residence times relative to other Greenland sites would provide valuable context. Shorter residence times, for example, could plausibly limit microbial methanogenesis and favour the export of thermogenic methane, a mechanism that would be consistent with the authors' interpretation and could be discussed more directly.
3. Role of subglacial and proglacial sediment
While the manuscript acknowledges the importance of sediment thickness for microbial methane production, it provides limited information on subglacial and proglacial sediment characteristics at the study site. Constraints on sediment thickness, organic carbon availability, bedrock type, or evidence for methanogenic microbial communities would help to better assess the relative importance of microbial versus thermogenic methane sources. Previous studies have shown that thick, organic rich subglacial sediments can substantially enhance methane production and export (e.g. Christiansen et al., 2021; Lamarche Gagnon et al., 2019), whereas thin or carbon poor sediments may strongly limit this process. Explicitly addressing these aspects, or clearly acknowledging current limitations, would strengthen the interpretation.
4. Potential contribution of groundwater springs
The possible contribution of groundwater pathways to methane export is not discussed in the current manuscript. Recent studies have demonstrated that proglacial groundwater springs emerging during glacier retreat can act as highly efficient conduits for methane release (e.g. Kleber et al., 2023). Clarifying whether such groundwater features are present, absent, or unlikely in the investigated catchment would help to better constrain the processes responsible for the observed methane signal.
5. Deglaciation history and permafrost aggradation
Beyond hydrological and sedimentary controls, the longer-term evolution of the study area may also influence methane production and export. The timing of deglaciation and subsequent permafrost aggradation could affect organic carbon availability and the development of subsurface methane reservoirs. Briefly integrating these processes into the discussion would provide a more comprehensive framework for interpreting the results, particularly in the context of ongoing and future glacier retreat.
Minor comments
• L106–107:
In Svalbard, methane has predominantly been measured in proglacial groundwater systems such as springs, often underlain by relatively young sedimentary bedrock. Kleber et al. (2023) focused specifically on groundwater springs, which are expected to exhibit much higher methane concentrations than surface meltwaters. As the present study primarily samples surface waters, lower methane concentrations are therefore expected. This paragraph would benefit from a clearer

distinction between sampling strategies and underlying processes.
• L173–174 and L179–180:
Please provide information on sediment thickness and organic carbon availability in subglacial and proglacial sediments in the study area. Thick, carbon rich sediments are likely to promote methanogenesis, whereas thin, carbon poor sediments may inhibit this process. Explicitly acknowledging the role of such conditions would strengthen the interpretation of methane fluxes.
• L187:
Could this statement be linked more directly to the authors' findings? Is there evidence in the dataset that surface waters exposed for longer periods following glacier retreat exhibit higher methane concentrations or fluxes?

Comments on figures
• Figure 1:
The study area map is difficult to interpret, whereas a clearer version is provided in the Supplementary Information. Two sites (26LAN1_2, 27LAN) are located on the ice sheet but are labelled as rivers. Revising the map for clarity and consistency would improve readability.
• Figure 2:
Data from Svalbard proglacial groundwater springs and recent studies from other mountain regions (e.g. Alpine catchments; Dalvai Ragnoli et al., 2025) appear to be missing. Including these datasets would strengthen the comparative context.
• Supplementary Figure 2:
The symbol used for "bitumen associated with mineral occurrences" is visually similar to that used for lake samples when printed at A4 scale. Revising the symbols would reduce the risk of misinterpretation. In addition, the point labelled 06LAN does not appear to correspond to a lake and should be corrected.
The manuscript addresses an important and timely question regarding CH_4 dynamics in glacial waters and is based on a valuable dataset; however, moderate revisions are required to strengthen the interpretation of results and better position the findings within the context of contemporary cryospheric research.

Best regards,
Reviewer

References
Christiansen, J.R., Röckmann, T., Popa, M.E., Sapart, C.J., Jørgensen, C.J., 2021. Carbon Emissions From the Edge of the Greenland Ice Sheet Reveal Subglacial Processes of Methane and Carbon Dioxide Turnover. Journal of Geophysical Research: Biogeosciences, 126(11): e2021JG006308.
Dalvai Ragnoli, M., Martini, J., Jechsmayr, B., Sapper, S.E., Singer, G., 2025. Probing dissolved CO2 and CH4 in glacial streams of the European Alps. Arctic, Antarctic, and Alpine Research, 57(1): 2580737.
Kleber, G.E. et al., 2023. Groundwater springs formed during glacial retreat are a large source of methane in the high Arctic. Nature Geoscience, 16(7): 597-604.
Lamarche-Gagnon, G. et al., 2019. Greenland melt drives continuous export of methane from the ice-sheet bed. Nature, 565(7737): 73-77.
Walter Anthony, K.M., Anthony, P., Grosse, G., Chanton, J., 2012. Geologic methane seeps along boundaries of Arctic permafrost thaw and melting glaciers. Nature Geoscience, 5(6): 419-426.

Version 1:

Reviewer comments:

Reviewer #2

(Remarks to the Author)
== General comments

I am glad to see that the authors have addressed all of my previous concerns, as well as I believe those of fellow reviewers. The revised manuscript is now much stronger and clearer, better contextualise the findings and I have now better confidence in their results and in the fact that they indeed show the presence of thermogenic methane in northern Greenland, as opposed to simply oxidised microbial methane (which to be clear still cannot be ruled out entirely by the data presented).

I therefore normally would not ask for additional revision. However, and I hate to have to insist on this point, I believe it is now essential for the authors to reference and slightly adjust part of their manuscript to reflect the results from a new study on Greenland methane that just came out in Nature Geosciences:

Hatton et al. 2026. Mid-Holocene retreat of the Greenland Ice Sheet indicated by subglacial methane release. https://doi.org/10.1038/s41561-026-01976-5.

To be clear, I don't think the new study by Hatton et al. 2026 impacts the relevance or the conclusion of the present study by Ketzer and colleagues. If anything I believe the new study makes the report of thermogenic glacial methane in northern Greenland even more important and relevant, as the study by Hatton et al. 2026 reports widespread occurrence of microbial methane released from the Greenland Ice Sheet from its entire Western margin (North to South).

However, this new dataset of Greenland methane from Hatton et al. 2026 should therefore be probably referenced and compared to. I believe the best way to do this in addition to added references in the text would be on Fig. 2. There is now glacial methane data from Northwest, Central West, and much more from South and South West Greenland that could easily be plotted on figure 2, probably keeping the same theme style but adding more Greenland Ice Sheet sectors accordingly. I think the Greenland-wide data might be better displayed as a range (e.g. vertical line for min/max) than simply mean given this is now clearly reported in Hatton et al. (I believe in one of the Extended data table). Minor point for Fig 2 as is, if the authors want to stick to also highlighting individual glaciers for Greenland, methane concentration for Russell Glacier should include data from Dieser et al. 2014, which showed much higher concentrations than reported. But I believe this is now too detailed with the new extended dataset from Hatton et al. and the authors here should derive the proper concentration ranges and means from these Greenland studies.

Another small point relating to the new study by Hatton et al. 2026 would be to also explicitly compare the findings from Northern Greenland with those from NW Greenland by Hatton et al. for likely cold-based glaciers there, which I think showed the lowest methane concentrations reported. Perhaps methane concentrations from meltwaters from cold-based glaciers could also be highlighted on a new Fig 2 to highlight the lower methane concentrations there but I'll leave this to the discretion of the authors as I appreciate the figure might become too busy.

== minor comments

Ln 25-27: I appreciate that the authors toned down their statement in specific response to one of my previous comment. I feel that perhaps the significance of the findings have now been toned down a bit too much. Perhaps re-phrasing to something like:

"These findings advance current understanding of methane sources beneath the Greenland Ice Sheet, revealing a thermogenic contribution in addition to previously reported microbial methane in Greenland meltwaters". Or something similar better, up to the authors if they want :).

Fig. 2 Add a reference to atmospheric equilibrium concentration. e.g. could be a horizontal line spanning the entire plot for a given water temp and salinity or a small horizontal line for each x sections (i.e. meltwaters, rivcers, lakes etc). This would very easily highlight that methane concentrations were almost always supersaturated.

Fig. 3 Some overlapping symbol - suggest using semi-transparent symbol if easily done by the authors. Also figure legend, change "periglacial" to "subglacial meltwaters". To me periglacial refers to areas next to a glacier but not necessarily connected to it.

Ln 161 and paragraph: For gas derived from the bitumen sample, it is explicitly stated that heavier hydrocarbons were present and measured in the sample, confirming the thermogenic origin of the methane for that sample. I suspect that a similar attempt was made for the other samples of dissolved methane but that the low concentrations of methane (and potentially ethane etc) prevented the detection of heavier hydrocarbons. If this is indeed the case that heavier hydrocarbons for the meltwater samples were below detection limit, it should at least be stated. Otherwise if these heavier hydrocarbons were detected, this in combination with the other evidence reported would help confirm a thermogenic origin. Similarly I think a line explicitly stating that deuterium stable isotopes could not be analysed due to the low methane concentrations of the samples should also be added to make clear to readers that this is the reason why the authors had to then only rely on 13C-CO2 and 13C-Ch4 for determining the origin of the methane, which is less robust and accurate the D and 13C-CH4 (as highlighted by the authors in their response to reviewer 1).

Supplementary Fig. 3 I think this new figure is a very nice addition and could stay as is. A small suggestion if easily done (and only a suggestion not binding to my review) would be to also add/overlay data by MacGregor 2022 to show which sector of the ice-sheet is likely cold-based, warm-based, or unknown. This would very nicely tie in the discussion on cold-based vs warm-based sites and highlight those on the map. I mostly recommend this given the authors already seem to be using GIS data from the MacGregor study and the paper is already cited by the authors:

MacGregor, J. A., Chu, W., Colgan, W. T., Fahnestock, M. A., Felikson, D., Karlsson, N. B., Nowicki, S. M. J., and Studinger, M.: GBaTSv2: a revised synthesis of the likely basal thermal state of the Greenland Ice Sheet, The Cryosphere, 16, 3033–3049, https://doi.org/10.5194/tc-16-3033-2022, 2022

Reviewer #3

(Remarks to the Author)
Dear Authors,
Thank you for the thorough revision. The manuscript has been substantially strengthened, particularly through a clearer and more rigorous interpretation of the dataset, improved positioning within current cryospheric CH_4 research, and resolution of previously noted ambiguities and inconsistencies. Overall, the revisions are convincing and fully address my comments, and I consider the manuscript suitable for publication and recommend acceptance.
Best regards,
Reviewer

Open Access This Peer Review File is licensed under a Creative Commons Attribution 4.0 International License, which permits use, sharing, adaptation, distribution and reproduction in any medium or format, as long as you give appropriate credit to the original author(s) and the source, provide a link to the Creative Commons license, and indicate if changes were made.
In cases where reviewers are anonymous, credit should be given to 'Anonymous Referee' and the source.
The images or other third party material in this Peer Review File are included in the article's Creative Commons license, unless indicated otherwise in a credit line to the material. If material is not included in the article's Creative Commons license and your intended use is not permitted by statutory regulation or exceeds the permitted use, you will need to obtain permission directly from the copyright holder.
To view a copy of this license, visit https://creativecommons.org/licenses/by/4.0/

Thermogenic methane beneath the northern Greenland Ice Sheet revealed by isotopic and geological evidence – Ketzer et al.

Point-by-point response to reviewers' comments (in red).

Reviewer #1 (Remarks to the Author):

Review of Nature Communications manuscript NCOMMS-26-019154
It is an excellent and well-written paper. The subject is highly topical and relevant. Knowing that it is from a remote and very difficultly accessible part of the world, the paper and as many background results as possible deserve to be published as soon as possible.

Response: We thank the reviewer for this very positive and supportive assessment.

In contrast to many other reviews, I only have a few direct suggestions, and I have only found a few errors or misunderstandings (marked in enclosed files).
I do not see any reason to be an anonymous reviewer; I was lucky enough to participate in field work and drilling in the region in 1984-85 and I was editing a large Bulletin summarizing all information of relevance to petroleum generation and accumulation in the Franklinian Basin (Christiansen ed. 1989). It is very interesting to see new gas data in a completely different context.
I have also been working in many other sedimentary basins in Greenland, and especially the Nuussuaq Basin in West Greenland where gas data may be of similar importance for discussions of biogenic versus thermogenic origin (Christiansen et al. 2020).
Both basins have been covered by ice and with low recent temperatures permafrost is many hundreds of meters thick.
Although it is important to discuss what is coming from below the Greenland Ice Sheet, it is for me similarly important to discuss what is coming from below the permafrost, and what was the timing of primary generation, later migration and release during uplift.
In the Franklinian Basin, the source rocks are very old – Cambrian and Silurian – and the organic material marine (no primary gas potential, gas is from later cracking of oil). Generation, migration and accumulation took place very early in history, probably during Devonian compression.
The Nuussuaq Basin is completely different with much younger source rocks – Cretaceous and Palaeogene – and with organic material including lots of terrestrial material that have been generating early gas. Generation was quick after loading of many km of volcanics. Large amounts of oil and gas are most likely still accumulated in the subsurface. Numerous visible seeps and core drilling suggest this.
These differences in timing clearly explain the very low concentration of methane in North Greenland compared to West Greenland. The isotope signatures are rather similar and in the thermogenic ranges. It is a pity that there are no $\delta DC1$ values that could give better information on thermal maturity and possible N-S variation.
The discussion in the paper would be even better if it included some comments on the generation and migration history, thermal maturity variation from North to South and

uplift history with comments on importance of permafrost and where the gas comes from.

Response: We thank the Reviewer for the positive and constructive assessment of our manuscript. We fully agree that explicitly addressing hydrocarbon generation, migration, and basin uplift provides an essential geological framework for interpreting the low methane concentrations observed in North Greenland. Incorporating these processes strengthens the discussion. Furthermore, expanding the discussion to include implications for other regions of Greenland, particularly West Greenland, broadens the relevance and impact of the manuscript. We agree with the Reviewer that the lack of δD measurements represents a limitation of the dataset. Unfortunately, δD analyses could not be conducted because methane concentrations were extremely low and sample volumes were prioritized for $\delta^{13}C$ analyses, precluding additional isotopic measurements. Regarding the Reviewer's comment on permafrost, we agree that this aspect is relevant to the discussion. We have therefore added a sentence addressing the potential role of permafrost, together with an appropriate reference, in the revised manuscript.

In light of the comments above, we have implemented the following revisions to the manuscript:

Action 1:
To address the low methane concentrations observed in North Greenland, we added the following text to the Discussion section (Lines 128-129):

"...early hydrocarbon migration during the Devonian, combined with effective degassing associated with major basin uplift during the Late Devonian to Early Carboniferous (370–350 Ma)..."

We also added information about the possible role of permafrost on gas migration (Lines 131-132):

"...existence of a thick permafrost acting as a seal for deep gas migration..."

These statements are supported by the following references, which are included in the revised manuscript:

Christiansen, F. G., Bojesen-Koefoed, J.A. Inventory of onshore petroleum seeps and stains in Greenland: a web-based GIS model. Geological Survey of Denmark and Greenland Bulletin 47 (2021). https://doi.org/10.34194/geusb.v47.6519

Christiansen, F.G., Koch, C.J.W., Nøhr-Hansen, H., Stouge, S., Thomsen, E. & Østfeldt, P. Petroleum Geology of North Greenland, Vol. 158 (ed. F.G. Christiansen), Chapter 6, 40–92 (Geological Survey of Greenland, 1989).

Higgins, A.K., Ineson, J.R., Peel, J.S., Surlyk, F. & Sønderholm, M. Lower Palaeozoic Franklinian Basin of North Greenland. Bulletin Grønlands Geologiske Undersøgelse 160, 71–139 (1991). https://doi.org/10.34194/bullggu.v160.6714

Action 2:

To expand the broader implications of our findings, we added the following text to the concluding part of the Discussion (Lines 222-225):

"In addition, our findings suggest that higher methane concentrations are more likely beneath the GrIS in regions hosting younger petroleum systems, such as the Nuussuaq Basin with Cretaceous and Palaeogene source rocks in West Greenland."

This statement is supported by the following reference, now included in the manuscript:

Christiansen, F.G., Bojesen-Koefoed, J.A., Dam, G., Laier, T. & Salehi, S. (2020). Oil and gas seepage in the Nuussuaq Basin, West Greenland – implications for petroleum exploration. GEUS Bulletin 44, 45–67. https://doi.org/10.34194/geusb.v44.4567

Specific revisions in response to Reviewer's comments annotated in the manuscript (pdf):

Main manuscript:
Figure 1: "RF" was replaced by "SF".
Lines 111–115: Text corresponding to Action 1 above was added.
Methods: We specified that gas analyses were conducted at Linnaeus University (Line 241).
References: Minor editorial corrections were made.

Supplementary Information:
Supplementary Figure 2 (caption): The term "mature" was replaced with "immature".
References: Minor editorial corrections were made.

Reviewer #2 (Remarks to the Author):

=== General comments

The manuscript by Ketzer et al. presents data on glacial methane from a very understudied and difficult to access area that is northern Greenland. Their results show small dissolved methane concentrations compared to other studies on glacial methane elsewhere in Greenland and other parts of the Arctic, but perhaps more importantly they attribute a thermogenic source of the methane detected based on stable isotope and geological data.

The presence of thermogenic methane in the area is not surprising given the overall geology and prior oil and gas prospective surveys in the area - as pointed out by the

authors - but confirming that thermogenic methane is indeed present from glacial sources in northern Greenland is important for future assessments of glacial methane emissions. I agree with the authors that this is much needed data for Greenland especially, and that this type of data can only be collected from very logistically challenging and costly field campaigns. Therefore I think that the data presented is of high relevance to the scientific community.

That being said, I think that some of the data interpretation and conclusions to be oversimplified based on the timing of the study and possible sampling locations which require further clarifications, and I am not convinced that the manuscript as currently written accurately contextualised the findings.

Response: We thank the reviewer for the constructive and thoughtful general comments on our manuscript, and for recognising the relevance and novelty of the data presented from North Greenland. We also acknowledge the reviewer's concerns regarding data interpretation, sampling context, and the need for clearer contextualisation of the results. We have carefully considered all points raised and have revised the manuscript accordingly. Each comment is addressed in detail below.

=== Major comments

1. Low methane concentrations and sampling context

Like the authors, I too was surprised about the very low methane concentrations reported for this area of Greenland given the likely thermogenic methane sources, especially when compared to other glaciated areas where thermogenic methane has been reported (e.g. Kleber 2023, 2025). However it is unclear whether these low methane concentrations are representative of concentrations expected to be found beneath the local glacial masses sourcing the sampled meltwaters, or artefacts of sampling locations, timing and seasonality. Samples were collected towards the very end of the melt season in late August/September, at time when I would expect minimal subglacial melt to be routed to the proglacial area. Studies on both Greenland and Svalbard (Lamarche-Gagnon 2019, Kleber 2025) show much lower glacial methane concentrations towards the end vs beginning of the melt-season, mostly due to a combination of increasing dilution of methane sources over the course of the meltseason, extensive flushing of the subglacial environment during the previous months, and meltwater shutdown towards the end of the meltseason. I would expect that for north Greenland, the timing of the study would even have a greater impact than let's say Svalbard given the colder temperatures and shorter melt-season. Perhaps more relevant is comparing the results here with those reported by Lamarche-Gagnon 2019 for late October meltwater samples in SW Greenland, which showed 1 order of magnitude smaller concentrations than during early and peak melt (i.e. ~18 nM vs 200 nM). These late-season SW Greenland concentrations are similar concentrations than those reported by the authors here.

I understand the logistical challenges that come with sampling that area of Greenland, that the authors were likely constrained by the timing of the cruise to collect those

samples, that earlier cruises are impractical to impossible in the region due to the presence of sea-ice and also that the many sites sampled by helicopter represent a true effort by the authors. The very high logistical challenges that come with collected these samples are one of the main reasons why these data are very valuable. But to me the reported methane concentrations in this study likely represent lower-concentration "end-members" from late melt-seasons at best.

Response: We agree with the Reviewer that the low methane concentrations measured in North Greenland can, at least in part, be attributed to a late melt-season sampling effect. This interpretation is supported by the seasonal framework described by Noël et al. (2022), who show that the melt season in North Greenland extends from April through September, placing our sampling at the very end of the melt season.

Action: We deleted the old sentence and added the following text to the discussion (Lines 133-137):

“The low methane concentrations measured in North Greenland can also be attributed to sampling at the end of the melt season, which in this region typically occurs in September (Noël et al., 2022). Consistent with this seasonal effect, late-season meltwater measurements from the southwestern GrIS show an approximately ten-fold decrease in methane concentrations relative to peak melt-season values, reaching levels comparable to those observed in this study (ca. 18 nM; Lamarche-Gagnon et al., 2019).”

References:

Lamarche-Gagnon, G., Wadman, J.L., Lollar, B.S., Arndt, S., Fietzek, P., Beaton, A.D., Tedstone, A.J., Telling, J., Bagshaw, E.A., Hawkings, J.R., Kohler, T.J., Zarski, J.D., Mowlem, M.C., Anesio, A.M., Stibal, M. Greenland melt drives continuous export of methane from the ice-sheet bed. Nature 565, 73-77 (2019). https://doi.org/10.1038/s41586-018-0800-0

Noël, B., Lenaerts, J. T. M., Lipscomb, W. H., Thayer-Calder, K., and van den Broeke, M. R.: Peak refreezing in the Greenland firn layer585 under future warming scenarios, Nature Communications, 13, 6870, https://doi.org/10.1038/s41467-022-34524-x

Moreover, more details regarding the exact sampling locations are needed in addition to only lat/lon coordinates (though those are of course needed). How were those "meltwaters" defined? It is very difficult from satellite images to assess how close to the glacial outlets the meltwater samples were collected and whether these meltwaters were exiting main subglacial water channels/outlets or consisted of lateral/periglacial meltwaters, which would be more diluted. I appreciate including photograph examples of some sampling site as supp material but I would add a photo for each sampling point if possible, especially for the meltwater sites. Even from the provided photographs, it is unclear what constitute the source of the samples waters. E.g. Supp Fig. 1d is labeled as meltwaters but no visible meltwater is present - perhaps maybe under the ice ledge but if so this further highlight my earlier points that late-season samples may not be

representative of true subglacial waters.

Further, can the author comment on whether the meltwaters originated from likely cold or warm-based glacial systems? It is my understanding that several of the smaller glacier in the area might be cold-based (unlike Petermann Glacier). I would not expect much methane to be flushed out from cold-based glaciers.

Response: We agree with the Reviewer that additional clarification regarding sample locations and water types would improve the manuscript. Accordingly, we now provide a clear definition of "meltwaters" and expand the documentation of sampling locations in both the main text and the Supplementary Information. In the revised manuscript, *meltwaters* are explicitly defined as waters flowing directly beneath the glacier, thereby distinguishing them from proglacial and periglacial surface waters. Running waters in the periglacial environment that are not directly connected to the subglacial system are consistently classified as rivers, creeks, or lakes. We believe this distinction is now clearly conveyed in the revised version. To further improve sampling location descriptions, we added in the Supplementary Information six new photographs illustrating representative sampling locations. The approximate position of each sampling point is now indicated by arrows directly on the photographs, improving spatial context. While additional photographs of other sampling environments (rivers, creeks, and lakes) can be provided if requested, we consider the newly added images sufficient to document the meltwater sampling strategy.

Regarding the basal thermal regime of glaciers, we address the Reviewer's comment by incorporating regional constraints from MacGregor et al. (2022). These data indicate that a large portion of North Greenland is cold-based, whereas Ryder and Petermann Glaciers are predominantly warm-based, and Ostenfeld Glacier exhibits a mixed warm- and cold-based configuration. We now explicitly acknowledge that the presence of cold-based glacier patches in North Greenland may further contribute to the low methane concentrations observed, due to limited basal meltwater production.
To address these points, we implemented the following specific changes:

Action 1: In the Introduction section describing sampling sites (Line 66), we added an explicit definition of meltwaters:

..."meltwaters (i.e., waters running directly underneath glaciers)..."

Action 2: We added six new photographs of additional meltwater sampling locations (Supplementary Figure 1).

Action 3: we added the following sentence to account for basal thermal conditions (Lines 132-133):

..." and the existence of cold-based glacier patches in North Greenland that limit basal meltwater production."

This interpretation is supported by the following reference, which has been incorporated into the revised manuscript:

MacGregor, J. A., Chu, W., Colgan, W. T., Fahnestock, M. A., Felikson, D., Karlsson, N. B., Nowicki, S. M. J., and Studinger, M. (2022) GBaTSv2: a revised synthesis of the likely basal thermal state of the Greenland Ice Sheet, The Cryosphere, 16, 3033–3049, https://doi.org/10.5194/tc-16-3033-2022, 2022

2. CO2 measurements and stable isotopes, and concomitant characterisation of thermogenic vs oxidised CH4sources.

I am also a bit concerned by the methods employed to measure dissolved CO2 and CO2 carbon stable isotopes; mainly the use of phosphoric acid. Essentially what the authors measured is total DIC as CO2 and not only dissolved CO2 concentrations (e.g. Salata et al. 2000). This method can have significant impact on the overall CO2 concentrations (e.g. Koschorreck et al. 2021), especially for waters with high alkalinity (which if often the case for glacial meltwaters). I believe this can be corrected by using pH and alkalinity but it is not clear from the methods that corrections were mades or that sample pH and alkalinity measured. Unless the authors can demonstrate or reference that 13C-DIC can be used instead of 13C-CO2 in stable-isotope genetic diagrams to infer methane gas sources.

The above does not completely discredit the results presented and the 13C-Ch4 remain the most important data to attribute a thermogenic methane origin. For what it's worth I still agree with the authors that a thermogenic origin for the methane is the most likely explanation and the authors present other lines of evidence to that regards (e.g. thermogenic methane from degassed limestone samples), but, given that determining whether or not the glacial methane detected is thermogenic is the key finding of the study, evidence needs to be presented as strongly as possible. Especially given that alteration of the stable isotopic signature by microbial oxidation cannot be systematically ruled out.

Response: We acknowledge the Reviewer's concern regarding the use of acid to degas samples and measure CO_2 concentration and stable carbon isotopes of the headspace. Our initial intention was to produce more headspace gas and improve the analyses. However, we fully agree with the Reviewer that methane genetic diagrams mostly use dissolved CO_2 for $\delta^{13}C$ isotopes and not the gas formed by the complete degassing of the sample (i.e., $\delta^{13}C$-DIC), with few exceptions (e.g., Avrahamov et al., 2010). Therefore, we decided to replace DIC with the original dissolved CO_2 data (concentration and isotopes) analysed from the headspace gas in equilibrium with water. Using CO_2 instead of DIC did not change the general interpretation of a thermogenic origin for methane, as also pointed out by the Reviewer, but caused a shift in the location of the sampling points with an enrichment in the light ^{12}C isotope in the genetic diagram (Figure 3). We believe that our isotopic argument is much stronger now. We thank the reviewer for this helpful comment, which led to an important improvement of the manuscript. The following actions were made to address reviewer's comments above:

Action 1: We replaced all references to DIC isotopic values with dissolved CO_2 data in the main text.

Action 2: We updated the genetic diagram (Figure 2) using the revised $\delta^{13}C\text{-}CO_2$ values.

Action 3: We revised the Methods section by replacing the DIC analytical description with the methodology used for CO_2 concentration and isotope measurements.

Reference:

Avrahamov et al. (2010) Characterization and Dating of Saline Groundwater in the Dead Sea Area. Radiocarbon, 52, p.1123-1140. https://doi.org/10.1017/S0033822200046208

3. Methane flux calculations

Whilst I commend the authors for their efforts in trying to calculate diffusive fluxes for their sampling sites and comparsion sites, given the very low concentrations reported and snapshot sampling, I believe these fluxes can difficulty be applied in a larger context, are highly speculative given the absence of data on river Q and v, and prevent any useful upscaling calculations and make the comparison with large world rivers not very relevant. I would therefore advise to remove the riverine methane flux comparison with other large rivers. I also have some reservations as to how these riverine fluxes were calculated (see specific comments below). I would simply keep comparisons between other glacial sites and potentially only for concentrations (Fig. 2a).

Response: We agree with the Reviewer that the methane flux data are of limited relevance to the present study and are not fully representative, as they rely on a single measurement at the low end-member range (end of melt season). In light of this, we recognize that these data may divert attention from the primary objective of the manuscript, namely the demonstration of thermogenic methane beneath the GrIS. Accordingly, we have followed the Reviewer's advice and removed the methane flux analysis from the manuscript. Comparisons of methane concentrations have been retained, as they support the discussion of methane origin. This revision shortens the manuscript by one paragraph and strengthens its focus on the central findings. The following actions were taken:

Action 1: Removal of discussion on methane flux.
Action 2: Removal of Figure 2b (including captions).
Action 3: Removal of methane flux calculations from the Methods.

== specific comments

Ln21: report measured concentration range

Response: Agreed and changed accordingly. Range values are now also reported on the Abstract (Line 21).

Ln25 & 35: I would tone down the language. "challenges" (abstract), "presumes" (Ln 35), etc. The findings are important and indeed previous reports in Western and Southern Greenland only attributed a microbial source for glacial methane, but I don't think any of these studies and follow-ups simply presumed that all glacial methane from Greenland would be strictly microbial, especially given widespread reports of thermogenic methane from glaciated catchments overlaying sedimentary rocks on Svalbard. In fact recent studies specifically allude to the fact that thermogenic methane is likely to be present in northern Greenland (e.g. Wadham et al. 2026). Therefore I would tone down the language a little bit and instead highlight the novelty of the findings as opposed to present those as a challenge to previous work.

Response: We agree with the Reviewer and have revised the text to place greater emphasis on the novelty of our findings. The revised sentence in the Abstract now reads (Lines 25-27):

"These findings extend current understanding of methane sources beneath the Greenland Ice Sheet by indicating a thermogenic contribution alongside microbial methane in Arctic methane cycling.".

In addition, we replaced "and assumes that" with "where" in the first paragraph of the Introduction to improve clarity and tone (Line 35).

Ln 41: Despite the somewhat provocative title by Christiansen 2018, the first direct observations of subglacial methane export in Greenland are from Dieser 2014 and Webster 2015. And technically the first "subglacial observations" could be attributed to deep ice core campaigns that measured elevated methane from basal ice in the 90s. I would therefore re-phrase to "first observations from subglacial meltwaters" and cite Dieser and Webster.

Response: We thank the Reviewer for drawing attention to these important early studies, and we have updated the sentence and added the two references accordingly (Lines 40-41). The new sentence is:

"Direct measurements of methane beneath the GrIS are still relatively recent, with first observations from subglacial meltwaters published just over a decade ago".

Ln42: "significant atmospheric emissions" This is still up for debate and no studies so far has directly measured or quantified atm emissions of glacial methane. If anything upscaling of diffusive riverine fluxes only amounts to modest emissions globally. I would tone down to something like "potentially significant" or "previously under appreciated"

Response: We agree with the Reviewer's suggestion and added "potentially" to that sentence (Line 42).

Ln40-54: I think these are nicely written paragraphs that present and introduce very well the relevance of the study.

Response: We thank the Reviewer for the positive and encouraging assessment of the Introduction and for highlighting the clarity and relevance of these paragraphs. No action required.

Ln54: Would also cite Svalbard work on glacial fracturing (Hodson 2020, Kleber 2023)

Response: The two references were added accordingly (Line 54).

Ln60: I would also add relevant to Canadian High Arctic, given the geographical proximity and geological similarities

Response: We added “Canadian High Arctic” according to the suggestion (Line 60).

Fig. 1. Nice map. 1b. I don't see any gas flare (yellow star) highlighted on the map, unlike stated in the legend. Also discrepancies between legend of Fig. S2 re lake vs meltwater sampling sites (e.g. near Peterman fjord). I would make sure all of those are consistent.

Response: The legend entry indicating gas flares (yellow star) was retained inadvertently from an earlier version of the map. This symbol has now been removed from the revised figure.

To resolve minor discrepancies between the two maps (and all figures), we improved visualization of sampling points to clearly indicate that two sampling locations are situated in close proximity. In addition, we changed the symbols of the corresponding sample in the geological map (Supplementary Figure 2) to ensure consistency with the location map (Figure 1) and methane genetic diagram (Figure 3). We expect that these revisions improve consistency and clarity across the figures.

Supp. Table 1: Add a column with sampling date. Also CO2 concentrations.

Response: We added sampling dates and carbon dioxide concentrations in Supplementary Table 1.

Ln104-116: see previous general comment re low concentrations likely attributed to seasonality (point 6) but perhaps more importantly sampling sites and "true" subglacial samples.

Response: We acknowledge the Reviewer’s comment and note that this issue has already been addressed through revisions made in response to Major Comment 1 (Low methane concentrations and sampling context).

Ln118-127: See previous general comment. I would keep the flux calculations to the Supp Material and avoid comparisons with large river systems.

Response: We acknowledge the Reviewer’s comment and note that this issue has already been addressed through revisions made in response to Major Comment 3

(Methane flux calculations). The methane flux discussions were removed from the manuscript (original lines 119-127 were removed).

Ln149: Is the gas-trapped from bitumen containing limestone CH_4 stable isotope plotted on Fig. 3? From the figure legend I would guess this is the red star on the figure but the stable istope signature plotted vs the one described in the text seem to differ slightly for $^{13}CO_2$ - i.e. -8.9 permil in the text vs less than -10 on the figure. This should be corrected, added, or clarified as I agree with the authors that this gas sample is probably one of the best end-member samples they have to compare their field methane samples and attribute to those a thermogenic origin.

Response: We thank the Reviewer for noting this issue. We identified that the isotopic value was incorrect in the text, while it was correctly reported in Figure 3 and Supplementary Table 1 (i.e., −14.2‰). The value has now been corrected in the text (Line 163).

Ln143-160: More information is needed to identify the samples/lakes connected or disconnected from glacial runoff. E.g. this could easily be highlighted in SuppTable 1. Same for creeks and rivers earlier in the text - how are those defined and are they completely disconnected hydrologically from glacial influence/runoff (except potentially from groundwaters)? Need more geographical description in the methods/SI and main text. Ln 159 seems to imply that lakes disconnected from glacial runoff are microbial - I would highlight on the figure.

Response: We agree with the Reviewer's comment and have explicitly identified in the text the two lakes that have no clear connection to glacial runoff, namely sites 05LAN and 08LAN (Line 177). This information has also been highlighted in Supplementary Figure 1: we replaced a photograph from the lake in site 09LAN with one from lake in site 08LAN to clear show no glacial influence and added the following text to the figure caption: "(c) lake in Wulff Land (site 08_LAN) with no clear connection to glacial runoff…". For comparison, we also added to the caption information about a lake with clear connection to glacier runoff (site 17LAN): "…(b) periglacial lake in Freuchen Land (site 17_LAN) with clear connection with glacial runoff…". The information was also highlighted in the caption of Figure 3, in line with the Reviewer's suggestion. The following text was added to the figure caption: The sample located within the "Primary microbial" field originates from a lake that shows no clear connection to glacial runoff (site 8LAN).

Response: In response to the Reviewer's comment regarding rivers and creeks, we added a brief explanation of their geographical context. All sampled rivers and creeks are hydrologically connected to glacial runoff, and this is now stated more explicitly in the manuscript. To clarify this point, we added the following sentence to the text (Lines 68-70):

"We also sampled waters of periglacial lakes (with and without clear connection with glacial runoff), creeks and rivers (all connected to glacial runoff), as well as an exposed limestone with oil stains for comparison (Fig. 1; Supplementary Figures 1 and 2)."

I also point out that only samples with highest methane concentrations from lakes could be unambiguously attributed to microbial methane. If possible I would add on the figure the expected fractionation trajectory from microbial oxidation. Given the very low methane concentrations of all samples, very difficult to trust at face value the absence of significant microbial oxidation influence.

Response: We acknowledge the point raised by the Reviewer and have added the methane oxidation trajectory in Figure 3. We added the following sentence to the caption: "The arrow indicates the methane oxidation trend".

Ln172 and main conclusion: I am not convinced by the data presented that northern Greenland/visited sites does not overlay larger thermogenic methane reserves, and that the low CH4 concentrations reported by the authors are not simple artefacts of sampling locations and time.

Response: We acknowledge the Reviewer's comment and recognize that the original wording may not have been sufficiently clear. Our intention was not to imply that large thermogenic methane reservoirs are absent in North Greenland. We aimed to convey that the presence of thermogenic methane–bearing bedrock alone is insufficient to predict high methane concentrations in overlying waters. To clarify this point, we have revised the sentence accordingly and added sampling locations and timing as additional factors (Lines 194-197):

"The comparatively low methane concentrations observed in northern Greenland suggest that methane storage beneath ice sheets cannot be inferred from bedrock type alone, and that additional factors, such as sampling location and timing relative to the melt season, may also exert a strong influence."

=== methods
Ln240-248: see previous comment regarding CO2 vs DIC measurements and acidification. Unless somehow CO2 concentrations were also derived and measured using the headspace from the CH4 vials and that bensalonium chloride does not significantly impact pH and that CO2 concentrations measure from the headsapce of the methane samples matched CO2 concentrations measured following acidification. Otherwise more details are needed to trust the 13C-CO2 measured following acidification.

Response: We agree that CO_2, rather than DIC, is the most appropriate parameter for methane genetic diagrams. Accordingly, we have replaced the $\delta^{13}C$-DIC data with the corresponding $\delta^{13}C$-CO_2 data. This issue has already been addressed through revisions made in response to Major Comment 2 (CO_2 measurements and stable isotopes. Specifically, we now use the isotopic values of CO_2 gas originally measured in unacidified water vials, rather than those obtained after acidification. All DIC data was removed from the revised manuscript.

Response: Regarding the comment on benzalkonium chloride, this compound exhibits near-neutral acid–base behaviour (Price, 1950; European Directorate for the Quality of Medicines & HealthCare, 2023). Consequently, no significant pH changes occur in our samples, especially given the low concentrations and small quantities applied.

References:

European Directorate for the Quality of Medicines & HealthCare. (2023). European Pharmacopoeia (11th ed., Monograph 0372: Benzalkonium chloride). Strasbourg, France: EDQM.

Price, P.B. (1950) Benzalkonium chloride (zephiran chloride) as a skin disinfectant. Arch Surg 61:23–33. https://doi.org/10.1001/archsurg.1950.01250020026004

ln257: CH_4 atm-eq concentration. Given the low dissolved CH4_aq measured by the authors, they should calculate a new CH_4 atm-eq concentration based on in situ water temp, salinities, but more importantly background atmospheric concentrations, which in 2024 should be (slightly) higher than in 2015 as per their reference.

Response: The discussion of methane flux was entirely removed from the revised manuscript following the Reviewer's recommendation in Major Comment 3 (Methane flux calculations). The associated methodological description was also removed accordingly. No further action is required.

Ln258: why 0.22C? is this the average water temperature for all sites? What was the temperature range?

Response: The methodological description for methane flux was also removed from the revised manuscript. See comment above. No further action is required.

Ln259: why were those stream velocities and slopes used? Was this based on any empirical/GIS data? The stream v seems high for this late period of the melt-season. Can the authors confirm high stream flow for all river/meltwater sites sampled? Doesn't look like it from photos (supp 2)

Response: The methodological description for methane flux was also removed from the revised manuscript. See comment above. No further action is required.

Ln269: how was water temperature measured?

Response: The methodological description for methane flux was also removed from the revised manuscript. See comment above. No further action is required.

Ln271: typo -> change Raynolds to Raymond et al.

Response: The methodological description for methane flux was also removed from the revised manuscript. See comment above. No further action is required.

Ln275-278: I now see the authors are basing their diffusive flux calculations based on LamarcheG-2019 which were calculated for a completely different meltwater-river system in South West Greenland that used in situ measurements and continuous river monitoring. River slope from 1 river-site to another one cannot simply be transposed. Ok to use broad boundary conditions to fork uncertainties but this should still be based on local physical conditions; slope can be quickly obtained from GIS for the areas in question. Or base those on global river surveys - e.g. Raymond 2012-13 model for low slope conditions

But perhaps more importantly and as discussed above, I don't think those flux calculations provide very useful data to contextualise the results.

Response: We agree with the Reviewer's comment and have therefore removed the methane flux calculations and associated discussion from the revised manuscript, following the recommendation in Major Comment 3 (Methane flux calculations). We believe that this revision has sharpened the focus of the manuscript and improved overall clarity.

We thank Reviewer #2 for the detailed review and valuable suggestions that have helped improve our work.

== References cited in the review

Kleber, G. E. et al. Proglacial methane emissions driven by meltwater and groundwater flushing in a high-Arctic glacial catchment. Biogeosciences 22, 659-674 (2025). https://doi.org:10.5194/bg-22-659-2025

Kleber, G. E. et al. Groundwater springs formed during glacial retreat are a large source of methane in the high Arctic. Nature Geoscience 16, 597-604 (2023). https://doi.org:10.1038/s41561-023-01210-6

Lamarche-Gagnon, G. et al. Greenland melt drives continuous export of methane from the ice-sheet bed. Nature 565, 73-77 (2019). https://doi.org:10.1038/s41586-018-0800-0

Koschorreck, M., Prairie, Y. T., Kim, J. & Marcé, R. Technical note: CO2 is not like CH4 – limits of and corrections to the headspace method to analyse pCO2 in fresh water. Biogeosciences 18, 1619-1627 (2021). https://doi.org:10.5194/bg-18-1619-2021

Hodson, A. J. et al. Sub-permafrost methane seepage from open-system pingos in Svalbard. The Cryosphere 14, 3829-3842 (2020). https://doi.org:10.5194/tc-14-3829-2020

Webster, K. D., White, J. R. & Pratt, L. M. Ground-Level Concentrations of Atmospheric Methane in Southwest Greenland Evaluated Using Open-Path Laser Spectroscopy and Cavity-Enhanced Absorption Spectroscopy. Arctic, Antarctic, and Alpine Research 47,

599-609 (2015). https://doi.org:10.1657/AAAR0014-051

Raymond, P. A. et al. Global carbon dioxide emissions from inland waters. Nature 503, 355-359 (2013). https://doi.org:10.1038/nature12760

Raymond, P. A. et al. Scaling the gas transfer velocity and hydraulic geometry in streams and small rivers. Limnology and Oceanography: Fluids and Environments 2, 41-53 (2012). https://doi.org:10.1215/21573689-1597669

Salata, G. G., Roelke, L. A. & Cifuentes, L. A. A rapid and precise method for measuring stable carbon isotope ratios of dissolved inorganic carbon. Marine Chemistry 69, 153-161 (2000). https://doi.org:https://doi.org/10.1016/S0304-4203(99)00102-4

Wadham, J. L. et al. An Ice Sheet-to-Ocean Analysis of Carbon Stores and Fluxes in Earth's Polar Regions (RECCAP2, Polar Ice Sheets). Global Biogeochemical Cycles 40, e2025GB008677 (2026). https://doi.org:https://doi.org/10.1029/2025GB008677

Reviewer #3 (Remarks to the Author):

General assessment
This manuscript addresses a timely and important topic: methane cycling in Arctic glacial catchments. The combined use of dissolved methane concentrations and stable isotope data in meltwaters is particularly valuable, as such datasets remain sparse in the literature. The study provides important evidence that thermogenic methane constitutes a substantial fraction of the exported methane signal, implying that continued glacier retreat may enhance fluxes of geologically sourced methane to the atmosphere. This finding is highly relevant in the context of glacier recession and is consistent with previous observations of cryosphere-controlled methane release pathways (e.g. Walter Anthony et al., 2012; Lamarche Gagnon et al., 2019).
Overall, the manuscript is clearly written and the conclusions are generally supported by the presented results. However, several aspects related to site representativeness, subglacial and proglacial context, and process interpretation would benefit from clarification. Addressing these points would strengthen the manuscript and improve its relevance for a broad readership. I therefore recommend moderate revision.

Response: We thank the Reviewer for the constructive and positive assessment of our manuscript. We appreciate the recognition of the value of the methane and stable isotope dataset and the relevance of our findings in the context of glacier retreat in Greenland. We agree that clarification of site representativeness, subglacial and proglacial context, and process interpretation would strengthen the manuscript. Accordingly, we have revised the text to address these points, with a particular focus on GrIS catchment characteristics, drainage and water residence time, subglacial sediments, and groundwater springs.

Major comments
1. Representativeness of the study site within the Greenland Ice Sheet (GrIS) context
The manuscript would benefit from a clearer positioning of the investigated catchment

relative to the Greenland Ice Sheet. It is not fully clear whether the study site is intended to represent a typical GrIS drainage system in northern Greenland, or whether it should instead be considered a glacierised basin adjacent to, but not fully representative of, the ice sheet. Providing additional information on the glaciological setting, including drainage area characteristics and its relation to the GrIS, would help readers better assess the broader applicability of the results. Alternatively, the discussion could more explicitly frame the findings in the context of glacierised basins outside the main ice sheet. Such clarification is particularly important given that subglacial sediment thickness beneath parts of the GrIS can be substantial (e.g. Lamarche Gagnon et al., 2019), whereas sediment properties at the study site are not clearly constrained.

Response: We thank the Reviewer for highlighting the need to more clearly position the investigated catchments relative to the GrIS. Our dataset includes samples from small basins adjacent to the main ice sheet, as well as from major drainage systems, including Peterman and C.H. Ostenfeld glaciers. Observations from these principal outlets, together with the known occurrence of the Franklinian Basin to the south, suggest that our data may be representative of a substantial portion of the northern GrIS. We have therefore added text to the Discussion to clarify this point (Lines 215-219):

"The extent to which these findings can be extrapolated across the northern sector of the GrIS remains poorly constrained. However, our observations from major drainage systems, including Peterman and C.H. Ostenfeld glaciers, together with the southward extent of petroleum systems within the Franklinian Basin (Supplementary Fig. 3), suggest that thermogenic methane may be distributed over a broad area beneath the northern GrIS."

To further clarify this point, we have included a new map showing the extent of the Franklinian Basin beneath the GrIS (Supplementary Figure 3).

2. Subglacial drainage and water residence time

Key aspects of the local subglacial hydrological setting, such as drainage efficiency and water residence time, are only briefly addressed in the manuscript, despite their central role in controlling methane production and export. A more explicit description, even at a qualitative level, of the expected drainage configuration and residence times relative to other Greenland sites would provide valuable context. Shorter residence times, for example, could plausibly limit microbial methanogenesis and favour the export of thermogenic methane, a mechanism that would be consistent with the authors' interpretation and could be discussed more directly.

Response: We concur with the Reviewer that the inclusion of brief qualitative context strengthens the discussion. Accordingly, we have added the following texts in two parts of to the manuscript (Lines 130-131):

..."possible shorter water residence time in the glacial environment of northern Greenland relative to southern GrIS basins (Förster et al., 2013; Miller et al., 2022)"...

And (Lines 171-174):

“...shorter water residence time in the subglacial environment (Förster et al., 2013; Miller et al., 2022), which limit the development of strongly reducing conditions required for methanogenesis and interaction times in cold subglacial environments where metabolic rates are inherently slow (Wadham et al., 2008; Yvon-Durocher et al., 2014; Conrad, 2023)”

References:

Conrad, R. Complexity of temperature dependence in methanogenic microbial environments. Frontiers in Microbiology 14, 1-13 (2023). https://doi.org/10.3389/fmicb.2023.1232946

Forster, R. R., Box, J.E., van den Broeke, Miège, C., Burgess, E.W., van Angelen, J.H., Lenaerts, J.T.M., Koening, L.S., Paden, J., Lewis, C., Gogineni, S.P., Leuschen, C., McConnell, J.R. Extensive liquid meltwater storage in firn within the Greenland ice sheet. Nature Geoscience 7, 95-98 (2013). https://doi.org/10.1038/ngeo2043

Miller, J. Z., Culberg, R., Long, D.G., Shuman, C.A., Schroeder, D.M., Brodzik, M.J. An empirical algorithm to map perennial firn aquifers and ice slabs within the Greenland Ice Sheet using satellite L band microwave radiometry. The Cryosphere 16, 103-125 (2022). https://doi.org/10.5194/tc-16-103-2022

Wadham, T., M., Tulaczyk, S., Sharp, M. Subglacial methanogenesis: A potential climate amplifier? Global Biogeochemical Cycles 22 (2008). https://doi.org/10.1029/2007GB002951

Yvon-Durocher, G., Allen, A.P., Bastviken, D., Conrad, R., Gudasz, C., St-Pierre, A., Thanh-Duc, N., del Giorgio, P.A. Methane fluxes show consistent temperature dependence across microbial to ecosystem scales. Nature 507, 488-491 (2014). https://doi.org/10.1038/nature13164

3. Role of subglacial and proglacial sediment
While the manuscript acknowledges the importance of sediment thickness for microbial methane production, it provides limited information on subglacial and proglacial sediment characteristics at the study site. Constraints on sediment thickness, organic carbon availability, bedrock type, or evidence for methanogenic microbial communities would help to better assess the relative importance of microbial versus thermogenic methane sources. Previous studies have shown that thick, organic rich subglacial sediments can substantially enhance methane production and export (e.g. Christiansen et al., 2021; Lamarche Gagnon et al., 2019), whereas thin or carbon poor sediments may strongly limit this process. Explicitly addressing these aspects, or clearly acknowledging current limitations, would strengthen the interpretation.

Response: We appreciate the Reviewer's comment and have expanded the discussion to better constrain the potential presence of organic-rich sediments beneath the GrIS. We now explicitly note that the absence of older sediments observed at our site is consistent with sediment cores from Sherard Osborn Fjord, where Holocene sediments lie directly on bedrock (O'Regan et al., 2021). Based on this regional context, we state that methane-generating, organic-rich sediments beneath the GrIS are unlikely, while acknowledging that their presence cannot be completely excluded.

Action: The following text was added to the revised manuscript (Supplementary Notes):

Bedrock and sediment cover in the study area

The bedrock consists of Neoproterozoic to Palaeozoic sedimentary successions of the Franklinian Basin, deposited in shelf and deep-water environments (Supplementary Figure 2). These units host petroleum systems with distinct source rocks, including (i) Cambrian carbonates and (ii) Silurian shales, with thermal maturity increasing northward from immature to post-mature and locally metamorphic conditions. The limestone sampled for comparative isotopic analyses belongs to the Hauge Bjerge Formation (Aeronian–Telychian, ~441–433 Ma) of the Peary Land Group.

Onshore sediments directly overlying the bedrock consist predominantly of coarse-grained material (gravel), occurring in subglacial settings as well as in lateral and frontal moraines. Finer-grained sediments are mainly associated with the proglacial environment, where glaciofluvial deposits include sand in addition to gravel, and lacustrine deposits are dominated by mud. Lacustrine deposits, in particular, have been cored during previous expeditions (Jakobsson et al., 2019) and consist primarily of light- to dark-gray silty clay with organic-rich intervals. Sediment thickness locally exceeds 8 m. However, these sediments are post-glacial (Holocene) in age (Jakobsson et al., 2019) and therefore do not contribute to methane generation beneath the Greenland Ice Sheet (GrIS). The absence of older sediments is also observed in sediment cores from Sherard Osborn Fjord, where Holocene sediments rest directly on bedrock (O'Regan et al., 2021). These observations suggest that thick, organic-rich sediments capable of contributing to methane generation beneath the GrIS in the study area are unlikely, although their presence remains uncertain.

References:
Jakobsson, M., Mayer, L., Farrel, J. SWEDARTIC Ryder 2019 Expedition Final Report. 456 (Luleå, 2019).

O'Regan, M., Cronin, T.M., Reily, B., Alstrup, A.K.O., Gemery, L., Golub, A., Mayer, L.A., Morlighem, M., Moros, M., Munk, O.L., Nilsson, J., Pearce, C., Detlef, H., Stranne, C., Vermassen, F., West, G., Jakobsson, M. The Holocene dynamics of Ryder Glacier and ice tongue in north Greenland. The Crysosphere 15, 4073-4097 (2021). https://doi.org/10.5194/tc-15-4073-2021

4. Potential contribution of groundwater springs
The possible contribution of groundwater pathways to methane export is not discussed

in the current manuscript. Recent studies have demonstrated that proglacial groundwater springs emerging during glacier retreat can act as highly efficient conduits for methane release (e.g. Kleber et al., 2023). Clarifying whether such groundwater features are present, absent, or unlikely in the investigated catchment would help to better constrain the processes responsible for the observed methane signal.

Response: We agree with the Reviewer that the manuscript would benefit from a brief discussion of groundwater springs as a potential source of methane-rich waters. However, no such features were observed in the study area. To address this point, we added the following clarification to the text (Lines 118-120): "...[waters] fed by groundwater springs can exhibit methane concentrations several orders of magnitude higher (up to 10^7 nM; Kleber et al., 2023). We did not observe such features in the study area."

Reference:

Kleber, G. E., Hodson, A.J., Magerl, L., Mannerfelt, E.S., Bradbury, H.J., Zhu, Y., Trimmer, M., Turchyn, A.V. Groundwater springs formed during glacial retreat are a large source of methane in the high Arctic. Nature Geoscience 16, 597-604 (2023). https://doi.org/10.1038/s41561-023-01210-6

5. Deglaciation history and permafrost aggradation
Beyond hydrological and sedimentary controls, the longer-term evolution of the study area may also influence methane production and export. The timing of deglaciation and subsequent permafrost aggradation could affect organic carbon availability and the development of subsurface methane reservoirs. Briefly integrating these processes into the discussion would provide a more comprehensive framework for interpreting the results, particularly in the context of ongoing and future glacier retreat.

Response: We thank the Reviewer for raising this point. We had already included a sentence on permafrost in response to a comment from Reviewer #1 (Lines 131-132), but we agree that the manuscript benefits from further clarification. We have therefore added the following sentences to the Discussion (Lines 200-210):

..."The expression of this microbial potential is modulated by hydrological residence times, which limit microbial processing under cold subglacial conditions, as well as by deglaciation history and permafrost evolution, which regulate the quantity and reactivity of organic carbon. Large permafrost-affected areas of the pan-Arctic shelf, for instance, contain organic carbon that is poorly reactive, thereby limiting microbial methanogenesis and increasing the relative contribution of deeper methane sources at the surface (Miesner et al., 2023). This framework is consistent with our observations, in which methane is predominantly derived from geological sources independently of subglacial biogeochemical processes. Further permafrost aggradation following glacier retreat may, however, form effective barriers to methane migration from depth (Christiansen et al., 2021), thereby reducing its expression at the surface."

Reference:

Miesner, F., Overduin, P.P., Grosse, G., Strauss, J., Langer, M., Westermann, S., von Deimling, T.S., Borvkin, V., Arndt, S. Subsea permafrost organic carbon stocks are large and of dominantly low reactivity. Scientific Reports 13, 9425 (2023). https://doi.org/10.1038/s41598-023-36471-z

Christiansen, F. G., Bojesen-Koefoed, J.A. Inventory of onshore petroleum seeps and stains in Greenland: a web-based GIS model. Geological Survey of Denmark and Greenland Bulletin 47 (2021). https://doi.org/10.34194/geusb.v47.6519

Minor comments

• L106–107:
In Svalbard, methane has predominantly been measured in proglacial groundwater systems such as springs, often underlain by relatively young sedimentary bedrock. Kleber et al. (2023) focused specifically on groundwater springs, which are expected to exhibit much higher methane concentrations than surface meltwaters. As the present study primarily samples surface waters, lower methane concentrations are therefore expected. This paragraph would benefit from a clearer distinction between sampling strategies and underlying processes.

Response: We agree with the Reviewer's comment and believe that this has been addressed in the Major comment 4 (Potential contribution of groundwater springs) above. Additional text can be added if necessary.

• L173–174 and L179–180:
Please provide information on sediment thickness and organic carbon availability in subglacial and proglacial sediments in the study area. Thick, carbon rich sediments are likely to promote methanogenesis, whereas thin, carbon poor sediments may inhibit this process. Explicitly acknowledging the role of such conditions would strengthen the interpretation of methane fluxes.

Response: We agree with the Reviewer's comment and believe that this has been addressed in the Major comment 3 (Role of subglacial and proglacial sediment) above. Additional text can be added is necessary.

• L187:
Could this statement be linked more directly to the authors' findings? Is there evidence in the dataset that surface waters exposed for longer periods following glacier retreat exhibit higher methane concentrations or fluxes?

Response: We thank the Reviewer for this comment. The statement in question is not derived from our own data, but from principles indicating that continued glacial retreat and associated isostatic uplift can reactivate geological structures (Olivier & Spada, 2015), potentially enhancing degassing from the bedrock. This process has been explicitly modelled by Vachon et al. (2022).

The direct connection to our data lies in the identification of methane of geological origin, most consistent with bedrock degassing, within the subglacial environment. We therefore consider this statement to represent an important implication of our findings, rather than a direct result, and believe it adds valuable context to the discussion. This is clearer now as we named the entire section “Implication”. We consider this statement an important implication of our findings but are happy to revise or remove it if deemed necessary.

References:

Olivieri, M., Spada, G. Ice melting and earthquake suppression in Greenland. Polar Science 9, 94-106 (2015). https://doi.org/10.1016/j.polar.2014.09.004

Vachon, R., Schmidt, P., Lund, B., Plaza-Faverola, A., Patton, H., Hubbard, A. Glacially induced stress across the Arctic from the Eemian interglacial to the present—Implications for faulting and methane seepage. Journal of Geophysical Research: Solid Earth 127, 1-38 (2022). https://doi.org/10.1029/2022JB024272

Comments on figures

• Figure 1:

The study area map is difficult to interpret, whereas a clearer version is provided in the Supplementary Information. Two sites (26LAN1_2, 27LAN) are located on the ice sheet but are labelled as rivers. Revising the map for clarity and consistency would improve readability.

Response: We agree that Figure 1 lacked clarity in its original form and have therefore improved the visualization of the sampling locations. We have also ensured that the same symbols are consistently used to represent the different sample types across all figures. We hope that these changes improve both clarity and coherence among the figures.

Sampling sites 26LAN2 and 27LAN are indeed creeks; however, they appear to overlie the ice sheet because the regional map lacks sufficient spatial resolution to resolve small creeks located between glaciers. To address the Reviewer’s comment, we have added the following clarification to the caption of Figure 1: “Note that sampling locations 26LAN2 and 27LAN, adjacent to Petermann Glacier, correspond to a lake and a creek, respectively. These features cannot be visually distinguished from the ice sheet at the map scale”.

• Figure 2:

Data from Svalbard proglacial groundwater springs and recent studies from other mountain regions (e.g. Alpine catchments; Dalvai Ragnoli et al., 2025) appear to be missing. Including these datasets would strengthen the comparative context.

Response: We agree with this comment and have added comparative data from the Eastern Alps (Ragnoli et al., 2025) and Svalbard (Kleber et al., 2023) to Figure 2. We believe that the inclusion of these datasets provides important wider context and

strengthens the interpretation of our samples. We changed the caption for Figure 2 accordingly.

• Supplementary Figure 2:
The symbol used for "bitumen associated with mineral occurrences" is visually similar to that used for lake samples when printed at A4 scale. Revising the symbols would reduce the risk of misinterpretation. In addition, the point labelled 06LAN does not appear to correspond to a lake and should be corrected.

Response: We agree with this comment and have revised the symbols across all figures. Symbols representing our samples are now shown in warm colours, while occurrences of bitumen and seeps in Supplementary Figure 2 are indicated using cold colours and distinct symbol shapes. As noted in our response to the comment on Figure 1, we have also ensured that the same symbols are used consistently to represent the different sample types throughout all figures. We hope that these revisions improve both the clarity and coherence of the visual presentation.

The manuscript addresses an important and timely question regarding CH_4 dynamics in glacial waters and is based on a valuable dataset; however, moderate revisions are required to strengthen the interpretation of results and better position the findings within the context of contemporary cryospheric research.

Response: We thank the Reviewer once again for constructive comments on our manuscript.

Marcelo Ketzer, corresponding author.
marcelo.ketzer@lnu.se

Thermogenic methane beneath the northern Greenland Ice Sheet revealed by isotopic and geological evidence – Ketzer et al.

Point-by-point response to reviewers' comments (in red).

Reviewer #2 (Remarks to the Author):

== General comments

I am glad to see that the authors have addressed all of my previous concerns, as well as I believe those of fellow reviewers. The revised manuscript is now much stronger and clearer, better contextualise the findings and I have now better confidence in their results and in the fact that they indeed show the presence of thermogenic methane in northern Greenland, as opposed to simply oxidised microbial methane (which to be clear still cannot be ruled out entirely by the data presented).

→ **Response:** We thank the reviewer for the careful reassessment and for the positive evaluation of the revised manuscript. We are pleased that the revisions have improved the clarity and contextualisation of our findings and strengthened confidence in the interpretation.

I therefore normally would not ask for additional revision. However, and I hate to have to insist on this point, I believe it is now essential for the authors to reference and slightly adjust part of their manuscript to reflect the results from a new study on Greenland methane that just came out in Nature Geosciences:

Hatton et al. 2026. Mid-Holocene retreat of the Greenland Ice Sheet indicated by subglacial methane release. https://doi.org/10.1038/s41561-026-01976-5.

To be clear, I don't think the new study by Hatton et al. 2026 impacts the relevance or the conclusion of the present study by Ketzer and colleagues. If anything I believe the new study makes the report of thermogenic glacial methane in northern Greenland even more important and relevant, as the study by Hatton et al. 2026 reports widespread occurrence of microbial methane released from the Greenland Ice Sheet from its entire Western margin (North to South).

However, this new dataset of Greenland methane from Hatton et al. 2026 should therefore be probably referenced and compared to. I believe the best way to do this in addition to added references in the text would be on Fig. 2. There is now glacial methane data from Northwest, Central West, and much more from South and South West Greenland that could easily be plotted on figure 2, probably keeping the same theme style but adding more Greenland Ice Sheet sectors accordingly. I think the Greenland-wide data might be better displayed as a range (e.g. vertical line for min/max) than simply mean given this is now clearly reported in Hatton et al. (I believe in one of the Extended data table). Minor point for Fig 2 as is, if the authors want to stick to also highlighting individual glaciers for Greenland, methane concentration for Russell Glacier should include data from Dieser et al. 2014, which showed much higher concentrations than reported. But I believe this is now too detailed with the new extended dataset from Hatton et al. and the authors here should derive the proper concentration ranges and means from these Greenland studies.

→ **Response**: We thank the reviewer for highlighting the relevant new study by Hatton et al. (2026). We have now incorporated and discussed this work at several points throughout the manuscript:

1. The broad representativeness and variability of methane concentrations reported by Hatton et al. (2026) are now acknowledged in the Introduction (Lines 45-46):

"A recent regional study encompassing most of western Greenland reported methane concentrations in subglacial and proglacial waters spanning four orders of magnitude (Hatton et al., 2026)."

2. A direct comparison between the values reported by Hatton et al. (2026) and those presented here has been incorporated into the manuscript (Lines 112-114):

" However, they are similar to some glaciers in the Northwest Greenland (Hatton et al., 2026) and exceed concentrations measured in southern Greenland, where values are near atmospheric equilibrium…".

3. The spatial distribution of thermogenic methane beneath the northern Greenland Ice Sheet is now better constrained through comparison with Hatton et al. (2026). The following sentence has been added to the Discussion (Lines 235-237):

"The western limit of thermogenic influence aligns with the margin of the Franklinian Basin, beyond which methane in the northwestern GrIS is primarily microbial (Supplementary Figure 3; Hatton et al., 2026)."

4. The location of the study sites by Hatton et al. (2026) are now shown in Figure 1.

5. Data from Hatton et al. (2026) have been incorporated into Figure 2.

We agree with the reviewer that presenting average, maximum, and minimum methane concentrations across different sectors of the GrIS is valuable. Accordingly, we have included these summary statistics for our dataset and for literature values in the caption of Figure 2.

We appreciate the reviewer's suggestion regarding the presentation of Figure 2. We have retained the glacier names in the figure, as they are directly referenced in the text (Lines 111-116 and) and facilitate a clearer connection between the discussion and the data presented. The dataset from Dieser et al. was not incorporated directly into Figure 2 because only methane concentration ranges are reported in the original study, which prevents consistent representation within the current plotting framework. Nevertheless, we recognise the importance of this study and have cited it at several points in the manuscript (e.g., Lines 41, 52, 214).

Another small point relating to the new study by Hatton et al. 2026 would be to also explicitly compare the findings from Northern Greenland with those from NW Greenland by Hatton et al. for likely cold-based glaciers there, which I think showed the lowest methane concentrations reported. Perhaps methane concentrations from meltwaters from cold-based glaciers could also be highlighted on a new Fig 2 to highlight the lower methane concentrations there but I'll leave this to the discretion of the authors as I appreciate the figure might become too busy.

→ **Response:** We thank the reviewer for raising this small but insightful point. We have now addressed the cold-based glacier aspect and compared to our findings by adding two sentences to the manuscript (Lines 135-136 and Lines 217-220):

"Cold-based glaciers are also present in northwest and south Greenland (MacGregor et al., 2022), where methane concentrations are similarly low (Hatton et al., 2026)."

"Meltwaters from cold-based glaciers in the North, Northwest, and South Greenland contain in general lower methane concentrations than the ones in Southwest and West Greenland (Hatton et al., 2026), which have warmer, thawed beds (MacGregor et al., 2022)."

→ We appreciate the reviewer's suggestion regarding additional information (cold-based glaciers) in Figure 2. While we agree that this information is valuable, we were concerned that incorporating it directly into the figure would reduce clarity and make it more difficult to interpret. To address the reviewer's suggestion while maintaining readability, we have instead included information on the basal thermal regime and methane concentrations in the figure caption, explicitly referring to selected glaciers shown in the figure (Lines 153-156):

"Notably, the lowest methane concentrations are associated with cold-based glaciers in the southern (e.g., Kiattuut Sermiat), northern (this study), and northwestern Greenland Ice Sheet, whereas the highest concentrations occur in the southwestern sector (e.g., Isunnguata Sermia), where glacier beds are predominantly thawed (MacGregor et al., 2022)."

== minor comments

Ln 25-27: I appreciate that the authors toned down their statement in specific response to one of my previous comment. I feel that perhaps the significance of the findings have now been toned down a bit too much. Perhaps re-phrasing to something like:

"These findings advance current understanding of methane sources beneath the Greenland Ice Sheet, revealing a thermogenic contribution in addition to previously reported microbial methane in Greenland meltwaters". Or something similar better, up to the authors if they want :).

→ **Response:** We agree with the comment by the reviewer and modified the sentence in the Abstract to enphasize a bit more our findings and keep an adequate tone. This is the new sentence (Lines 25-27):

"These findings advance current understanding of methane sources beneath the Greenland Ice Sheet, revealing a thermogenic contribution alongside microbial methane in Arctic methane cycling."

Fig. 2 Add a reference to atmospheric equilibrium concentration. e.g. could be a horizontal line spanning the entire plot for a given water temp and salinity or a small horizontal line for each x sections (i.e. meltwaters, rivcers, lakes etc). This would very easily highlight that methane concentrations were almost always supersaturated.

→ **Response:** We agree with the reviewer that adding a line for the atmospheric equilibrium concentration would facilitate the interpretation of the figure. A dashed line was added together with the following explanation in the caption (Lines 159-160):

"The dashed line indicates the atmospheric equilibrium concentration of methane.".

Fig. 3 Some overlapping symbol - suggest using semi-transparent symbol if easily done by the authors. Also figure legend, change "periglacial" to "subglacial meltwaters". To me periglacial refers to areas next to a glacier but not necessarily connected to it.

→ **Response:** We agree with the suggestion and made the symbols semi-transparent and replaced "periglacial" with "subglacial metlwaters" in the caption.

Ln 161 and paragraph: For gas derived from the bitumen sample, it is explicitly stated that heavier hydrocarbons were present and measured in the sample, confirming the thermogenic origin of the methane for that sample. I suspect that a similar attempt was made for the other samples of dissolved methane but that the low concentrations of methane (and potentially ethane etc) prevented the detection of heavier hydrocarbons. If this is indeed the case that heavier hydrocarbons for the meltwater samples were below detection limit, it should at least be stated. Otherwise if these heavier hydrocarbons were detected, this in combination with the other evidence reported would help confirm a thermogenic origin. Similarly I think a line explicitly stating that deuterium stable isotopes could not be analysed due to the low methane concentrations of the samples should also be added to make clear to readers that this is the reason why the authors had to then only rely on 13C-CO2 and 13C-Ch4 for determining the origin of the methane, which is less robust and accurate the D and 13C-CH4 (as highlighted by the authors in their response to reviewer 1).

→ **Response:** We agree with the reviewer that it is important to clearly state that heavier hydrocarbons were below the detection limit. This information was already included when presenting methane concentrations across the different samples (Lines XX: "no heavier hydrocarbons were detected"). However, we agree that the manuscript benefits from reiterating this point in the context of the isotopic measurements of the bitumen sample. Accordingly, we have revised the relevant sentence to make this clearer (Lines 176-179):

"Despite the absence of measurable heavier hydrocarbons in meltwater and periglacial stream samples, their isotopic signatures indicate a predominantly thermogenic methane source, and/or potential influence of oxidation processes."

→ We also agree with the comment on deuterium stable isotopes and have now added a new sentence to the Methods (Lines 297-299):

"The δD_{CH4} measurements could not be conducted because methane concentrations were extremely low and sample volumes were prioritized for $\delta^{13}C_{CH4}$ analyses."

Supplementary Fig. 3 I think this new figure is a very nice addition and could stay as is. A small suggestion if easily done (and only a suggestion not binding to my review) would be to also add/overlay data by MacGregor 2022 to show which sector of the ice-sheet is likely cold-based, warm-based, or unknown. This would very nicely tie in the discussion on cold-based vs warm-based sites and highlight those on the map. I mostly recommend this given the authors already seem to be using GIS data from the MacGregor study and the paper is already cited by the authors:

MacGregor, J. A., Chu, W., Colgan, W. T., Fahnestock, M. A., Felikson, D., Karlsson, N. B., Nowicki, S. M. J., and Studinger, M.: GBaTSv2: a revised synthesis of the likely basal thermal

state of the Greenland Ice Sheet, The Cryosphere, 16, 3033–3049, https://doi.org/10.5194/tc-16-3033-2022, 2022

→ **Response:** We thank the reviewer for recognising the value of this figure and suggesting its inclusion in the manuscript. We agree that incorporating the thermal state of the Greenland Ice Sheet bed is an excellent idea. Accordingly, we have added the distribution of cold (frozen) and warm (thawed) basal conditions based on MacGregor et al. (2022). This addition enhances the figure, making it more informative and increasing its relevance to the study.

→ We sincerely thank the reviewer for this second revision and for the constructive feedback, which has contributed to improving the manuscript.

Reviewer #3 (Remarks to the Author):

Dear Authors,
Thank you for the thorough revision. The manuscript has been substantially strengthened, particularly through a clearer and more rigorous interpretation of the dataset, improved positioning within current cryospheric CH_4 research, and resolution of previously noted ambiguities and inconsistencies. Overall, the revisions are convincing and fully address my comments, and I consider the manuscript suitable for publication and recommend acceptance.
Best regards,
Reviewer

→ We thank the reviewer for the positive assessment and for the constructive comments provided throughout the review process. We are pleased that the revisions have addressed the concerns and improved the clarity and positioning of the manuscript.
